# Energy Efficient Single Pulse Switching of [Co/Gd/Pt]_N_ Nanodisks Using Surface Lattice Resonances

**DOI:** 10.1002/advs.202204683

**Published:** 2022-12-11

**Authors:** Maxime Vergès, Sreekanth Perumbilavil, Julius Hohlfeld, Francisco Freire‐Fernández, Yann Le Guen, Nikolai Kuznetsov, François Montaigne, Gregory Malinowski, Daniel Lacour, Michel Hehn, Sebastiaan van Dijken, Stéphane Mangin

**Affiliations:** ^1^ Université de Lorraine Institut Jean Lamour UMR CNRS 7198 Nancy 54011 France; ^2^ Department of Applied Physics Aalto University School of Science P.O. Box 15100 Aalto FI‐00076 Finland; ^3^ Department of Materials Science and Engineering and Department of Chemistry Northwestern University Evanston Illinois 60208 USA

**Keywords:** all‐optical magnetization switching, plasmonics, surface lattice resonance, ultrafast physics

## Abstract

The impact of plasmonic surface lattice resonances on the magneto‐optical properties and energy absorption efficiency has been studied in arrays of [Co/Gd/Pt]_N_ multilayer nanodisks. Varying the light wavelength, the disk diameter, and the period of the array, it is demonstrated that surface lattice resonances allow all‐optical single pulse switching of [Co/Gd/Pt]_N_ nanodisk arrays with an energy 400% smaller than the energy needed to switch a continuous [Co/Gd/Pt]_N_ film. Moreover, the magneto‐optical Faraday effect is enhanced at the resonance condition by up to 5,000%. The influence of the disk diameter and array period on the amplitude, width and position of the surface lattice resonances is in qualitative agreement with theoretical calculations and opens the way to designing magnetic metasurfaces for all‐optical magnetization switching applications.

## Graphical abstract

This work aims to propose a new approach for energy efficient ultrafast magnetic recording in magnetic nanodisks. In this perspective, it is shown that the excitation of plasmonic collective resonances, so‐called surface lattice resonances, in magnetic metasurfaces allows to reduce by 400% the threshold energy for single pulse all‐optical magnetization switching combined with a 5,000% enhancement of magneto‐optical readout sensitivity.

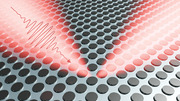

## 1 Introduction

All‐optical magnetization manipulation using ultrashort laser pulses and its potential applicability fits with the ceaseless demand for ultrafast and energy efficient magnetic recording.^[^ ^1^, ^2^, ^3^, ^4^, ^5^, ^6^, ^7^, ^8^, ^9^, ^10^, ^11^, ^12^, ^13^, ^14^, ^15^, ^16^, ^17^ ^]^ Single pulse all‐optical helicity‐independent switching (AO‐HIS) has been demonstrated first in GdFeCo alloys in 2011.^[^ ^4^ ^]^ AO‐HIS is believed to be a thermal effect that induces the demagnetization of the Gd and FeCo sublattices whose demagnetization timescales are different and allow exchange of angular momentum between Gd and FeCo leading to toggle switching.^[^ ^3^, ^4^ ^]^ Compared to hard‐disk drives on the market, the writing speed of AO‐HIS in ferrimagnetic rare‐earth transition‐metal (RE‐TM) alloys is ≈ 10 times faster with a full magnetization switching time around hundreds of ps.^[^ ^4^, ^8^ ^]^ Recently, it has been reported that single pulse AO‐HIS is also achievable in Co/Gd multilayers^[^ ^18^ ^]^ with timescales comparable to the corresponding RE‐TM alloys.^[^ ^19^ ^]^ This system is of particular interest because the AO‐HIS can be achieved without any composition requirements^[^ ^18^, ^20^ ^]^ in opposite to GdFeCo alloys or Tb/Co multilayers,^[^ ^21^ ^]^ facilitating the production on wafers. The fluence needed to switch magnetization with one single laser pulse is much lower than the other systems showing AO‐HIS.^[^ ^17^, ^18^ ^]^ Moreover this synthetic‐ferrimagnetic multilayer fits the demands of the data storage industry as it can overcome thermal annealing^[^ ^22^ ^]^ required for fabricating nanostructures or opto‐controllable magnetic tunnel junctions.^[^ ^23^, ^24^ ^]^

In future applications, AO‐HIS is needed to achieve ultrafast magnetic recording while increasing current all‐optical areal recording densities requires nanostructures whose writing resolution is not limited by the diffraction limit. Plasmonics provide the tools to manipulate light beyond the diffraction limit.^[^ ^25^ ^]^ In 2015, Liu et al. exploited two‐wire plasmonic gold nanoantennas on a TbFeCo film to induce all‐optical switching in an area whose lateral size is 53 nm with a threshold fluence reduction of 37% thanks to field enhancement in the near field.^[^ ^13^ ^]^ Moreover Kataja et al. showed in 2018 that demagnetization and field‐assisted magnetization switching in periodic magnetic nanoparticles are improved at the surface lattice resonance (SLR) wavelength due to the enhanced nanoparticle absorption facilitated by these modes.^[^ ^26^ ^]^

In this paper, we study plasmon‐assisted femto‐second laser‐induced single pulse AO‐HIS and the magneto‐optical response of [Co/Gd/Pt]_N_ nanodisk arrays. The effect of the disk diameter, the array period and the light wavelength on optical switching and the magneto‐optical response are determined. We demonstrate that at the SLR condition the energy needed to switch [Co/Gd/Pt]_N_ nanodisks arrays by one single laser pulse is drastically reduced. We also present extinction calculations that are in good agreement with our experimental data.

## 2 Results and Discussion

**Figure 1:**
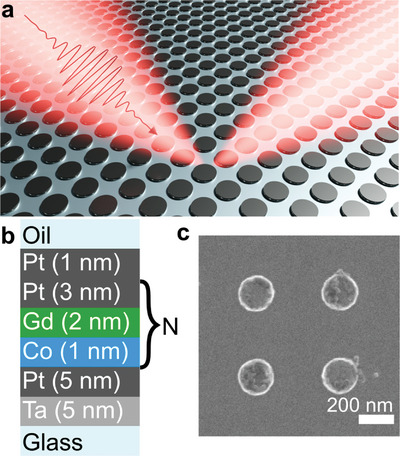
Magnetic [Co/Gd/Pt]_N_ metasurface. a) Schematic view of the AO‐HIS. b) Sketch of the [Co/Gd/Pt]_N_ multilayer stack. c) SEM image of a [Co/Gd/Pt]_N_ nanodisk array with *D* = 200 nm and *P* = 500 nm.

**Figure** **1**a shows a sketch of AO‐HIS in a magnetic metasurface consisting of a square array of [Co/Gd/Pt]_N_ nanodisks. The nanodisk arrays are embedded in index‐matching oil (*n* = 1.5) on a glass substrate to ensure long‐range coupling between them resulting in SLR.^[^ ^27^ ^]^ As presented in Figure 1b, the nanodisks consist of a Pt(1)/[Pt(3)/Gd(2)/Co(1)]_N_/Pt(5)/Ta(5) multilayer stack, with the numbers in parenthesis indicating the layer thickness in nanometer and *N* the number of repetitions. Hereafter, we will refer to the magnetic nanodisk array as [Co/Gd/Pt]_N_ metasurface. The nanodisk arrays were prepared using a top–down approach involving electron‐beam lithography, lift‐off, and Ar ion beam etching (see Figure S1, Supporting Information). The optical and magneto‐optical properties of the [Co/Gd/Pt]_N_ metasurfaces depend on the diameter of the nanodisks (*D*) and the period of the square array (*P*). Here, we report the influence of nanodisk diameter (*D* = 150, 200, and 250 nm) and array period (*P* = 500 and 550 nm) on single‐pulse AO‐HIS. Scanning electron microscopy (SEM) (Figure 1c) measurements on a metasurface with *D* = 200 nm, *P* = 500 nm, and *N* = 2 are shown as an example of the fabricated metasurfaces. Microscopy images of other samples are shown in Figure S2 (Supporting Information).

We measured the hysteresis loops of the [Co/Gd/Pt]_N_ nanodisk arrays and the unpatterned [Co/Gd/Pt]_N_ film with *N* = 1, 2, 3, 4, 5, and 6 using the polar magneto‐optical Faraday and Kerr effect, respectively (see Figure S4, Supporting Information). The coercive field of the films ranges from 9.5 to 3.2 mT for *N* = 1, 2, 3, and 4. For *N* > 4, the remanence decreases significantly because of domain formation (see Figure S4, Supporting Information). From MFM images and the domain size in [Co/Gd/Pt]_N = 5,6_ continuous films, we deduce that the [Co/Gd/Pt]_N = 5,6_ nanodisks are still single domain. Besides, the perpendicular magnetization of [Co/Gd/Pt]_N_ metasurfaces reverses abruptly in an applied magnetic field for any *N*. Magnetic switching in the [Co/Gd/Pt]_N_ nanodisks requires larger magnetic field than the unpatterned film due to the lower probability of domain nucleation within the disks.

**Figure 2:**
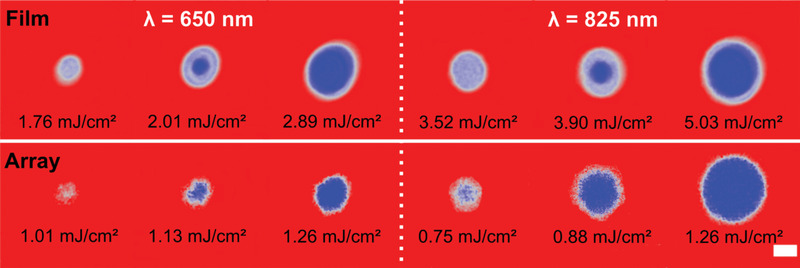
Single‐pulse AO‐HIS for a continuous [Co/Gd/Pt]_2_ film (top panels) and for a patterned [Co/Gd/Pt]_2_ metasurface with *D* = 150 nm and *P* = 500 nm (bottom panels) at *λ* = 650 nm, and *λ* = 825 nm. Red and blue colors indicate magnetization pointing up and down, respectively. The laser fluence is indicated in the figure. The scale bar corresponds to 50 µm.

**Figure 3:**
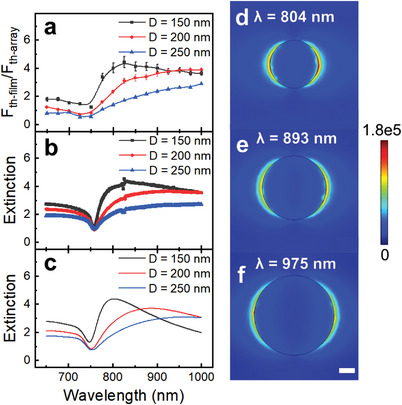
a) Ratio (*F*_*th* − *film*_/*F*_*th* − *array*_) between the AO‐HIS threshold fluence measured on a [Co/Gd/Pt]_2_ film and on corresponding metasurfaces with *P* = 500 nm and different nanodisk diameters. b) Extinction spectra of the [Co/Gd/Pt]_2_ metasurfaces normalized to their filling factor and the extinction spectrum of the [Co/Gd/Pt]_2_ continuous film. c) Simulated normalized extinction spectra for the same metasurfaces. d–f) Distribution of electric field intensity (|*E*|/|*E*_0_|) in a square array of nanodisks with *P* = 500 nm at the SLR wavelength. The diameter of the disks is d) 150, e) 200, and f) 250 nm. The scale bar corresponds to 50 nm.

We focused on *N* = 2 for studying the impact of plasmon excitations on single pulse AO‐HIS as a good compromise between strong magneto‐optical signal and achievable toggle switching in the continuous film at any wavelength of interest for AO‐HIS. Experiments with linearly polarized light, along one of the primary axes of the nanodisk arrays, were conducted at wavelengths ranging from 650 to 1000 nm. The AOS experiments were conducted using 216 fs laser pulses from a Yt fiber laser with regenerative amplifier (see Figure S3, Supporting Information). Images of magnetization switching obtained on the [Co/Gd/Pt]_2_ film and a [Co/Gd/Pt]_2_ metasurface with *D* = 150 nm and *P* = 500 nm are presented in **Figure** **2** for different laser fluences and for a wavelength of 650 and 825 nm. By comparing the size of the switched area and the fluence needed to achieve AO‐HIS, it is obvious that the metasurface switches at considerably smaller energy than the unpatterned film. While more efficient AO‐HIS is attained in the metasurface at both wavelengths, the effect is particularly strong for 825 nm, where the laser fluence is reduced by a factor 4. The wavelength dependence of single‐pulse AO‐HIS in metasurfaces with *D* = 150, 200, and 250 nm and *P* = 500 nm and the unpatterned film is summarized in **Figure** **3**a (see Figure S5, Supporting Information, for *P* = 550 nm). Here, we plot the ratio of the laser threshold fluence needed to induce optical switching in the film and metasurface (*F*_*th* − *film*_/*F*_*th* − *array*_). At almost all wavelengths, single‐pulse AO‐HIS is more efficient in the metasurfaces than the unpatterned film and the condition of optimal switching efficiency shifts to larger wavelength with increasing nanodisk diameter.

The improved energy efficiency of AO‐HIS in the metasurfaces is explained by the excitation of a collective SLR, as suggested by the optical extinction curves shown in Figure 3b. The extinction spectra are shaped by the diffracted order (DO) of the nanodisk array (sharp minimum at ≈ 760 nm) and a broader SLR (maximum following the DO) resulting from hybridization between the DO and the local surface plasmon resonance of individual nanodisks.^[^ ^28^, ^29^, ^30^, ^31^ ^]^ Since the SLR mode absorbs light more than it scatters^[^ ^32^, ^33^ ^]^ the extinction spectra are a good measure of light absorption by the [Co/Gd/Pt]_2_ metasurfaces (see Figure S6, Supporting Information, for spectra of other metasurfaces with different *N*). As AO‐HIS is driven by a pure thermal effect, strong optical absorption by the SLR explains the improved energy efficiency of AO‐HIS in the metasurfaces. To corroborate that stronger optical absorption per unit area explains the gain in switching efficiency, we scaled the extinction spectra of the metasurfaces with their filling factor (area covered by the nanodisks divided by the total area) and normalized the result to the extinction recorded on the continuous film (Figure 3b). Clearly the shapes of the normalized extinction curves closely resemble the threshold fluence data shown in Figure 3a. This correspondence provides a powerful tool for minimizing the AO‐HIS energy in magnetoplasmonic systems through straightforward extinction measurements using a continuous laser. Finite element method (FEM) simulations of the normalized extinction (Figure 3a) support the experimental findings. For the metasurfaces discussed here (*P* = 500 nm), most efficient AO‐HIS is attained for the metasurface with *D* = 150 nm because of the strongest optical absorption per unit area, as further confirmed by the simulated intensity of the optical near fields shown in Figure 3d–f (see Figure S7, Supporting Information, for the optical constants).

**Figure 4:**
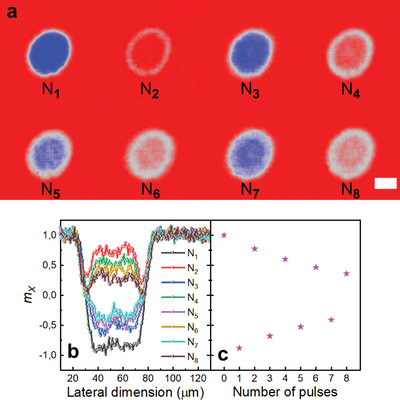
a) MOKE images after each single pulse switching for a [Co/Gd/Pt]_2_ metasurface with *D* = 150 nm and *P* = 500 nm. The laser fluence is 1.26 mJ cm^−^^2^ and the wavelength is at 650 nm. b) Corresponding switching rate profiles across the switching areas after each single‐pulse switching experiment. c) Switching rate at the center of the metasurface as a function of the number of pulses. The scale bar indicates 50 µm.

To further support the conclusions on AO‐HIS, we performed repetitive switching measurements on the continuous films and metasurfaces.^[^ ^34^ ^]^ While the films show 100% repetitive single pulse AO‐HIS (see Figure S8, Supporting Information), the switching rates are smaller for the corresponding metasurfaces. For instance, **Figure** **4**a depicts 8 MOKE images recorded following repetitive switching by eight successive laser pulses for *D* = 150 nm, *P* = 500 nm, and laser fluence *F* = 1.26 mJ cm^−^^2^. The switching rate, defined as $m\;=\;\frac{M}{M_{0}}$ decreases when the disk diameter increases (see Figure S8, Supporting Information), reaching 88% for *D* = 150 nm, 76% for *D* = 200 nm, and 66% for *D* = 250 nm. In particular, we notice that the switching rate *m_X_* after *X* pulses can be written as

(1) $$m_{X}=\prod_{i}m_{i}\;\;with\;\;\sum i=\;X$$

as confirmed by Figure 4b showing the normalized radial switching rate profiles after 8 successive laser pulses. Then Figure 4c plots the switching rate at the center of the metasurface as a function of lase pulse number illuminating the metasurface. We conclude from Figure 4 and Figure S8 (Supporting Information) that the switching rate increases when the disk size decreases, paving the path to reach 100% repetitive switching for magnetic bits even smaller than 150 nm.

**Figure 5:**
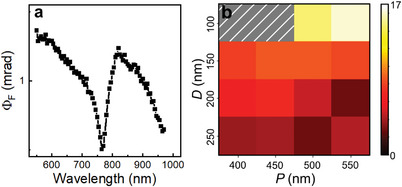
Faraday readout sensitivity. a) Faraday angle spectrum for a [Co/Gd/Pt]_2_ metasurface with *D* = 150 nm and *P* = 500 nm. b) Ratio of the Faraday angles measured on the [Co/Gd/Pt]_2_ metasurfaces and the [Co/Gd/Pt]_2_ continuous films at the SLR wavelength, scaled to the filling factors of the nanodisks arrays.

The data presented thus far demonstrate how SLR excitations can significantly aid the writing process in all‐optical magnetic recording. Magneto‐optical readout of stored information is another key requirement of this data storage technology. To assess the effect of collective SLRs on the magneto‐optical read‐out sensitivity of [Co/Gd/Pt]_N_ metasurfaces, we measured the magneto‐optical Faraday angle Φ_F_ as a function of nanodisk diameter *D*, array period *P*, and repetition number *N* ^[^ ^35^ ^–^ ^38^ ^]^ (see Figure S9, Supporting Information). **Figure** **5**a shows the Faraday angle spectrum for a metasurface with *D* = 150 nm and *P* = 150 nm. The measurement demonstrates that the Faraday angle is minimal at the DO ≈ 760 nm and that the SLR mode increases the magneto‐optical signal. The latter effect is explained by a resonant enhancement of the magneto‐optical activity when the inverse polarizability of the individual [Co/Gd/Pt]_N_ nanodisks matches the lattice factor of the periodic array.^[^ ^35^, ^38^ ^]^ To quantify the magneto‐optical readout sensitivity, we extracted the ratio of the Faraday angles of the [Co/Gd/Pt]_N_ metasurfaces and of the [Co/Gd/Pt]_N_ continuous films at the SLR wavelengths, and we scaled this ratio by the filling factors of the nanodisk arrays.^[^ ^37^ ^]^ Figure 5b summarizes the magneto‐optical readout‐sensitivity for [Co/Gd/Pt]_2_ metasurfaces (see Figure S10, Supporting Information). When readout is performed locally, an increase of the magneto‐optical output signal by up to a factor 15 is achieved for *N* = 2, while the enhancement factor is ≈ 50 for *N* = 6 (see Figure S10, Supporting Information). Also, we note that the SLR enhances the magneto‐optical response most for *D* = 100 or 150 nm because of the more intense optical near‐fields in smaller nanodisks (see Figure 3d–f).

**Figure 6:**
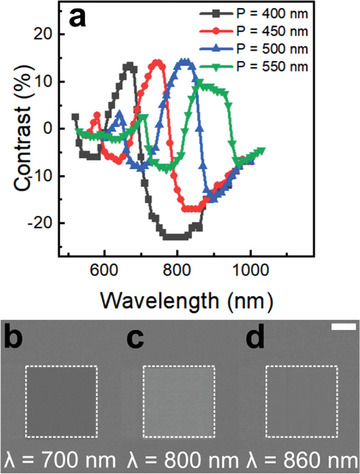
Magneto‐optical contrast of [Co/Gd/Pt]_3_ metasurfaces. a) Magneto‐optical contrast for *D* = 150 nm and *P* = 400, 450, 500, and 550 nm. b–d) $\frac{\Delta I}{\Sigma I}\;$for *P* = 500 nm with specific insets at 700, 800, and 860 nm respectively. The scale bar corresponds to 100 µm.

Another parameter that is essential for magneto‐optical read‐out is the signal‐to‐noise ratio (SNR), which relates to the magneto‐optical contrast at the readout wavelength. Indeed, we had to optimize this quantity when performing ultrafast measurements in a reflection configuration (see Figure S3, Supporting Information) as all the statements about AO‐HIS are based on images analysis. To quantify the SNR, we define the magneto‐optical contrast as

(2) $$C\;=\;\;\frac{\Delta I}{\Sigma I}=\frac{I_{0+}-I_{0-}}{I_{0+}+I_{0-}}\;$$

where *I*_0 +_ (*I*_0 −_ ) is the light intensity probed for magnetization pointing up (down). Δ*I* and Σ*I* are linked to the Kerr rotation *θ* and ellipticity *η* so that the contrast can be rewritten as

(3) $$C\;=\;\;\frac{\Delta I}{\Sigma I}=\frac{\sin\left(2\alpha \right)\times \theta }{\sin{\left(\alpha \right)}^{2}+\left(\theta^{2}+\eta^{2}\right)\times \cos\left(\alpha \right)}\;$$

where *α* is the angle between the polarizer and the analyzer. Modulation of the Kerr rotation and ellipticity by the excitation of a SLR provides accurate tuning of the wavelength at which the SNR is maximized. **Figure** **6** depicts how the magneto‐optical contrast shifts when the array period is changed for a metasurface with *D* = 150 nm (see Figure S11, Supporting Information).

## 3 Conclusion

In summary, we demonstrated energy‐efficient plasmon‐assisted magnetization switching and sensitive magneto‐optical readout in perpendicularly magnetized metasurfaces comprising periodic arrays of [Co/Gd/Pt]_N_ nanodisks. Both the single‐pulse optical writing and magneto‐optical readout are significantly enhanced by the excitation of collective SLRs. The laser threshold fluence for magnetization switching and the magneto‐optical response of the metasurfaces can be accurately tuned by the disk diameter and array period providing a versatile design strategy towards the realization of energy‐efficient plasmon assisted magnetic recording technologies.

## 4 Experimental Section

### Film Growth

The Pt(1)/[Pt(3)/Gd(2)/Co(1)]_N_/Pt(5)/Ta(5) multilayer stack was grown on a glass substrate by magnetron sputtering in a PVD‐8 system from Vinci Technologies. The Ar deposition pressure was set to 5  ×  10^−8^ Torr and all layers were grown at room temperature.

### Metasurface Fabrication

The [Co/Gd/Pt]_N_ metasurfaces were patterned from the continuous multilayer stack using e‐beam lithography in a RAITH 150–2 system. In the lithography process, a 50 nm thick Al etching mask was first defined using a lift‐off process. Hereafter, ion beam etching (IBE) was used at an optimized angle of 10° in a 4wave system to pattern the multilayer stack into periodic nanodisk arrays with different disk diameters and array periods. Finally, the Al layer was removed by a wet etching step. The metasurface fabrication process is illustrated in Figure S1 (Supporting Information).

### AOS Measurements

AOS experiments on continuous [Co/Gd/Pt]_N_ film and [Co/Gd/Pt]_N_ metasurfaces were conducted with a Yt fs fiber laser with regenerative amplifier and optical parametric amplifier (OPA). The wavelength of the writing pulse was varied from 650 to 1,000 nm in 25 nm steps. The writing pulse was linearly polarized along one on the primary axes of the square nanodisk array and at normal incidence. The duration of the writing pulse was fixed at 216 fs and its repetition rate was 100 kHz. The spot size of the writing pulse was estimated as 225 µm (using the 1/e^2^ convention). The magnetic switching effect was imaged with at different wavelengths to maximize the SNR.

### Optical and Magneto‐Optical Measurements

A magneto‐optical spectrometer was used to measure the optical transmission and magneto‐optical Faraday effect as a function of wavelength from 550 to 1,000 nm. The setup pictured in Figure S3 (Supporting Information) consisted of a broadband supercontinuum laser (SuperK EXW‐12 from NKT Photonics), polarizing and focusing optics, a photoelastic modulator (Hinds Instruments I/FS50), and a photodetector. The transmission and magneto‐optical spectra were recorded at normal incidence. The Faraday rotation (*θ*) and Faraday ellipticity (*ε*) were simultaneously recorded by lock‐in amplification of the modulated signal at 50 and 100 kHz. From these data, the magneto‐optical Faraday angle (Φ) was calculated using $\Phi =\sqrt{\theta^{2}+\eta^{2}}$.

### Simulations

Optical transmission (extinction) spectra were calculated by finite element method (FEM) simulations in COMSOL software. A unit cell with one nanodisk was simulated. Periodic boundary conditions were applied at the edges of the unit cell to simulate the electric‐field distribution of square nanodisk arrays. The mesh consisted of free triangular elements with a maximum size of 2 nm. All simulations were conducted with linearly polarized incident light along the sample normal with a wavelength ranging from 650 to 1,000 nm. A uniform embedding medium with a dielectric constant of *n* = 1.5 was used. The refractive index dataset was measured as shown on Figure S20 (Supporting Information). In particular, the optical spectra of the metasurfaces and the continuous films were simulated as presented in Figure 3c while Figure 3d–f plot the near‐field distribution in the plane of nanodisks of different sizes.

### Ellipsometry Measurements

The sample with linearly polarized light was illuminated and the change of polarization was probed upon reflection from the sample to obtain the complex reflectance ratio ****ρ**** = ***r***_***p***_/***r***_***s***_. Optical constants were extracted from the ellipsometry measurements using CompleteEASE software and assuming a single layer system. The CompleteEASE software utilizes a Bruggeman effective medium approach to minimize the mean‐square deviation between measured and calculated ellipsometry parameters. The extracted effective refractive index was used in COMSOL simulations to calculate transmission (1 – extinction) and reflection spectra as well as the distribution of optical near‐fields.

## Conflict of Interest

The authors declare no conflict of interest.

## Author Contributions

M.V., S.P., J.H., S.V.‐D., and S.M. planned the study. M.H. grew the multilayer stack. M.V. fabricated the metasurfaces by e‐beam lithography and conducted scanning electron microscopy measurements. S.P. and Y.L.‐G. performed the optical transmission and magneto‐optical measurements. M.V. and J.H. conducted AOS experiments and Y.L.‐G. assisted the measurements. M.V., S.P., and Y.L.‐G. analyzed the optical, magneto‐optical and AOS data with input from other authors. M.V. performed the FEM simulations. M.V., J.H., S.V.‐D., and S.M. wrote the manuscript with inputs from all authors. All authors discussed the results.

## Supporting information

Supporting Information

## Data Availability

The data that support the findings of this study are available from the corresponding author upon reasonable request.
