# Supplementary material for: Energy Efficient Single Pulse Switching of [Co/Gd/Pt]N Nanodisks Using Surface Lattice Resonances
Source: Adv Sci (Weinh). 2022 Dec 11;10(4):2204683. doi: 10.1002/advs.202204683 (PMC9896076; doi:10.1002/advs.202204683)

ADVANCED SCIENCE
Open Access

Supporting Information

for *Adv. Sci.*, DOI 10.1002/advs.202204683

Energy Efficient Single Pulse Switching of $[Co/Gd/Pt]_N$ Nanodisks Using Surface Lattice Resonances

Maxime Vergès, Sreekanth Perumbilavil, Julius Hohlfeld, Francisco Freire-Fernández, Yann Le Guen, Nikolai Kuznetsov, François Montaigne, Gregory Malinowski, Daniel Lacour, Michel Hehn, Sebastiaan van Dijken and Stéphane Mangin**

Supporting Information

Energy efficient single pulse switching of [Co/Gd/Pt]$_N$ nanodisks using surface lattice resonances

Maxime Vergès, Sreekanth Perumbilavil, Julius Hohlfeld, Francisco Freire-Fernández, Yann Le Guen, Nikolai Kuznetosv, François Montaigne, Gregory Malinowski, Daniel Lacour, Michel Hehn, Sebastiaan van Dijken and Stéphane Mangin**

S1. Fabrication process

Figure S1 details the steps for obtaining periodic arrays of nanodisks from the full multilayer stack.

S2. Microscopy analysis of [Co/Gd/Pt]$_N$ metasurfaces

Figures S2, S3, S4, S5, S6, and S7 provide SEM images for metasurfaces with N = 1, 2, 3, 4, 5, and 6 respectively.

S3. Experimental setups

Figures S8 and S9 illustrate the setups for AOS experiments and optical and magneto-optical measurements, respectively.

S4. Magnetic properties

Figures S10 and S11 present the magnetic hysteresis loops that were measured using the magneto optical Kerr effect for the [Co/Gd/Pt]$_N$ continuous films and metasurfaces, respectively. Figure S12 shows MFM measurements of [Co/Gd/Pt]$_N$ continuous films for N = 5 and 6.

S5. AO-HIS experiments on [Co/Gd/Pt]$_2$ metasurfaces

Figure S13 shows similar results as in Figure 3 for P = 550 nm.

S6. Extinction spectra of the [Co/Gd/Pt]$_N$ metasurfaces

Figures S14, S15, S16, S17, S18, and S19 provide extinction spectra of the [Co/Gd/Pt]$_N$ metasurfaces.

S7. Optical constants of [Co/Gd/Pt]$_N$ continuous films

Figure S20 presents measured optical constants of [Co/Gd/Pt]$_N$ continuous films.

S8. Repetitive switching

Figures S21, S22 and S23 provide repetitive switching images data for a [Co/Gd/Pt]$_2$ continuous film, a metasurface with D = 200 nm and P = 500 nm and a metasurface with D = 250 nm and P = 500 nm, respectively.

S9. Magneto-optical spectra of the [Co/Gd/Pt]$_N$ metasurfaces and continuous films

Figures S24, S25, S26, S27, S28, and S29 present magneto-optical spectra of the [Co/Gd/Pt]$_N$ metasurfaces while figure S30 provides the optical and magneto-optical spectra for the continuous films.

S10. SLR-enhanced Faraday read-out sensitivity

Figure S31 summarizes the SLR enhanced Faraday effect for [Co/Gd/Pt]$_N$ metasurfaces.

S11. Magneto-optical contrast

Figure S32 shows magneto-optical contrast spectra of [Co/Gd/Pt]$_3$ metasurfaces for D = 200 nm and 250 nm.

Figure S1. Lithography steps in the fabrication process of $[Co/Gd/Pt]_N$ metasurfaces**.**

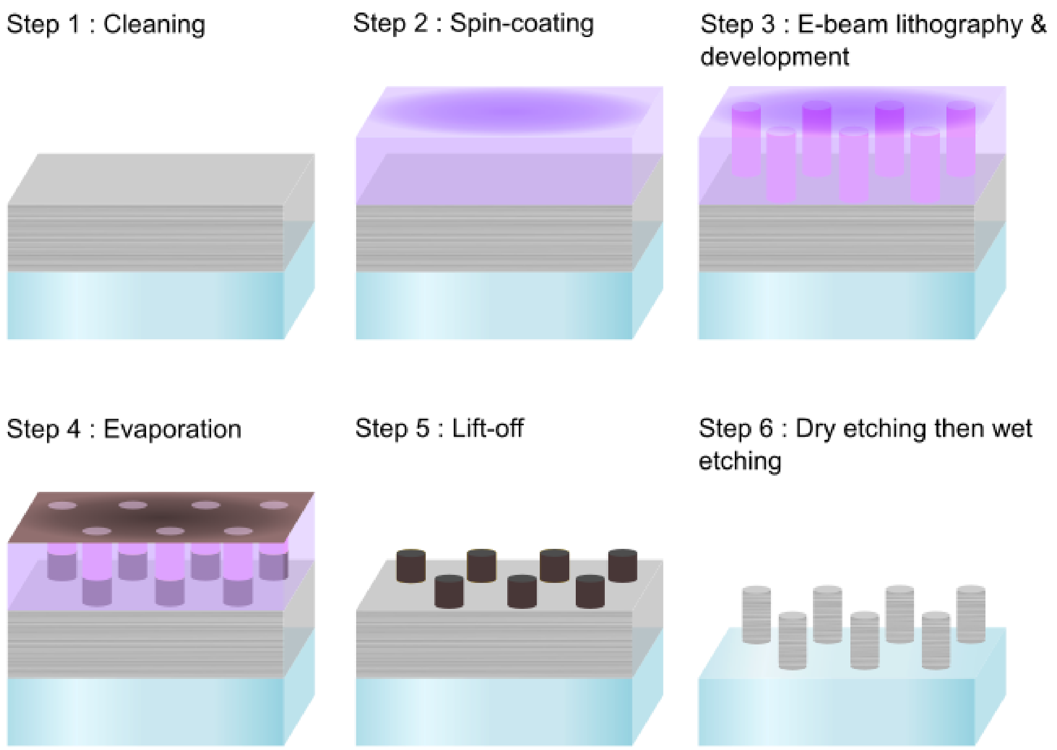

Figure S2. SEM images of $[Co/Gd/Pt]_1$ nanodisk arrays. The scale bar corresponds to 200 nm.

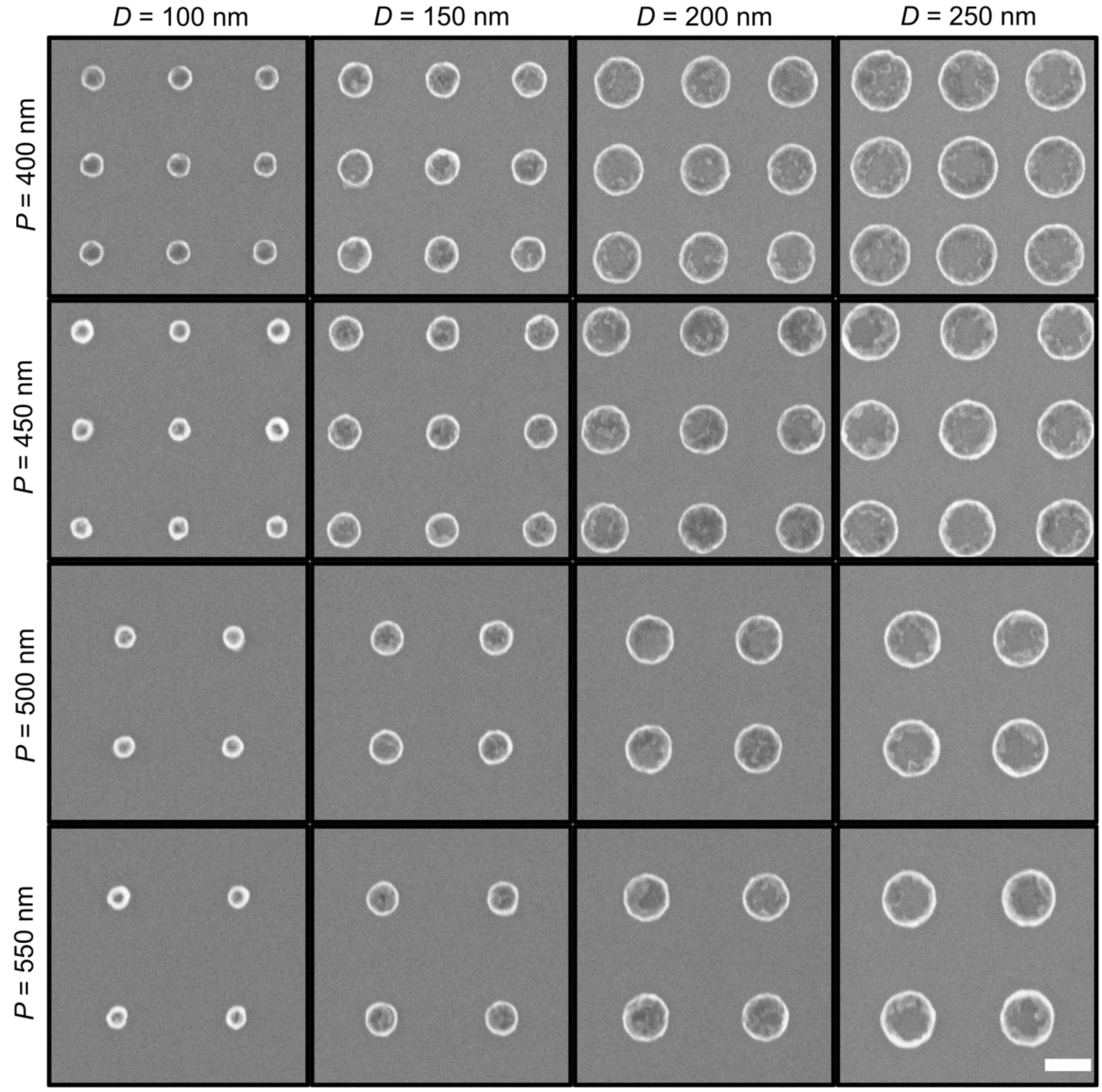

Figure S3. SEM images of $[Co/Gd/Pt]_2$ nanodisk arrays. The scale bar corresponds to 200 nm.

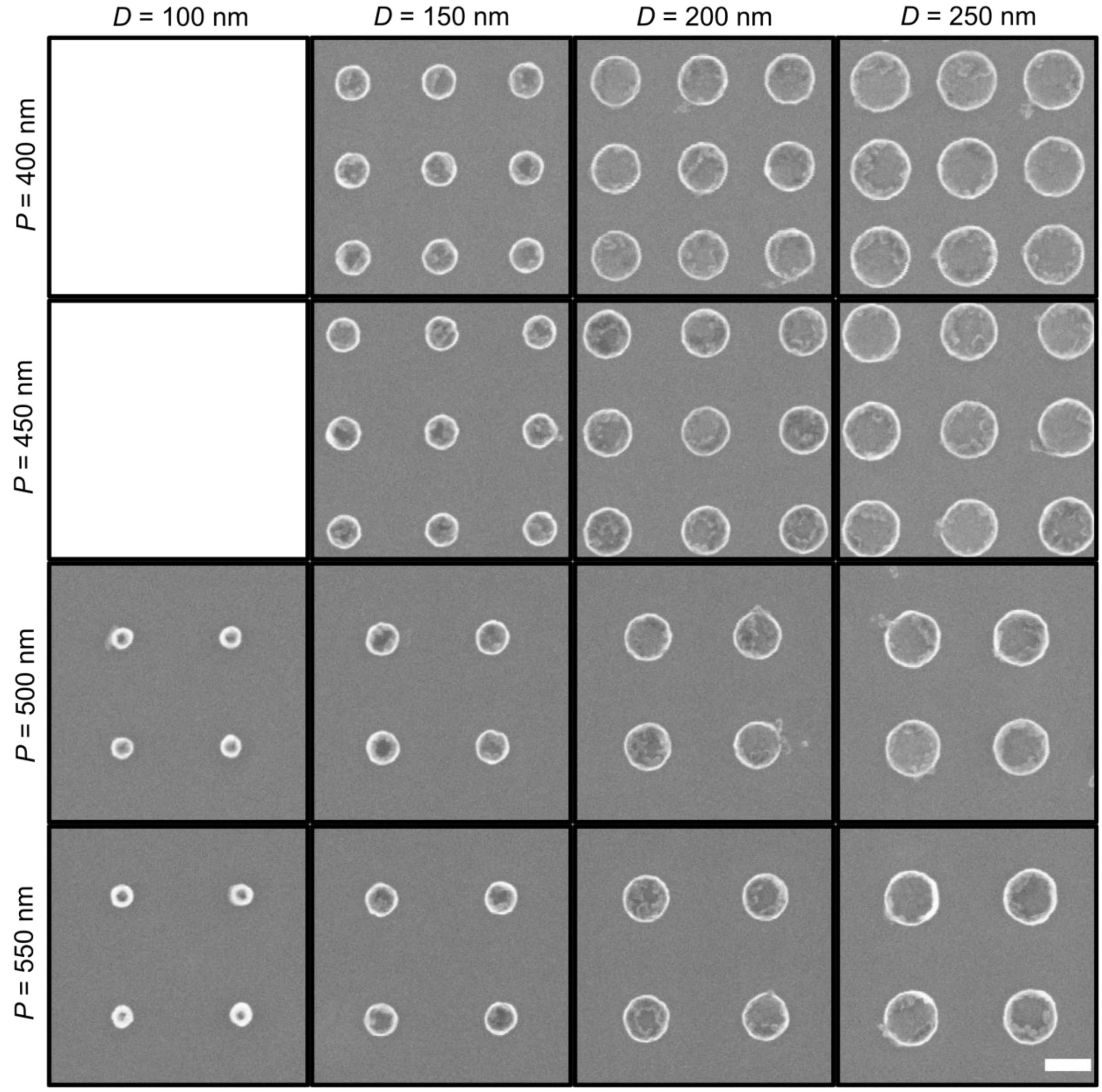

Figure S4. SEM images of $[Co/Gd/Pt]_3$ nanodisk arrays. The scale bar corresponds to 200 nm.

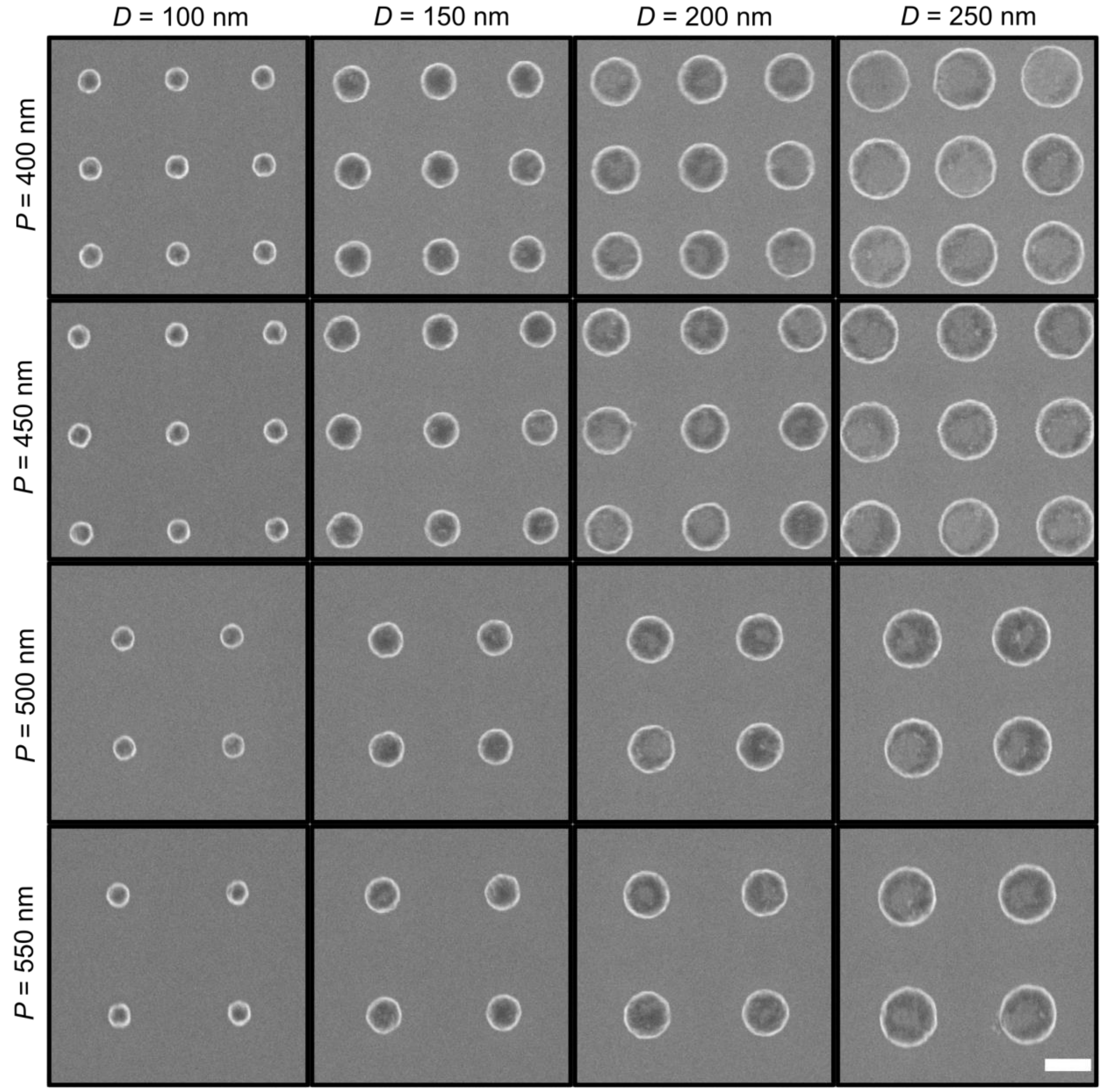

Figure S5. SEM images of $[Co/Gd/Pt]_4$ nanodisk arrays. The scale bar corresponds to 200 nm.

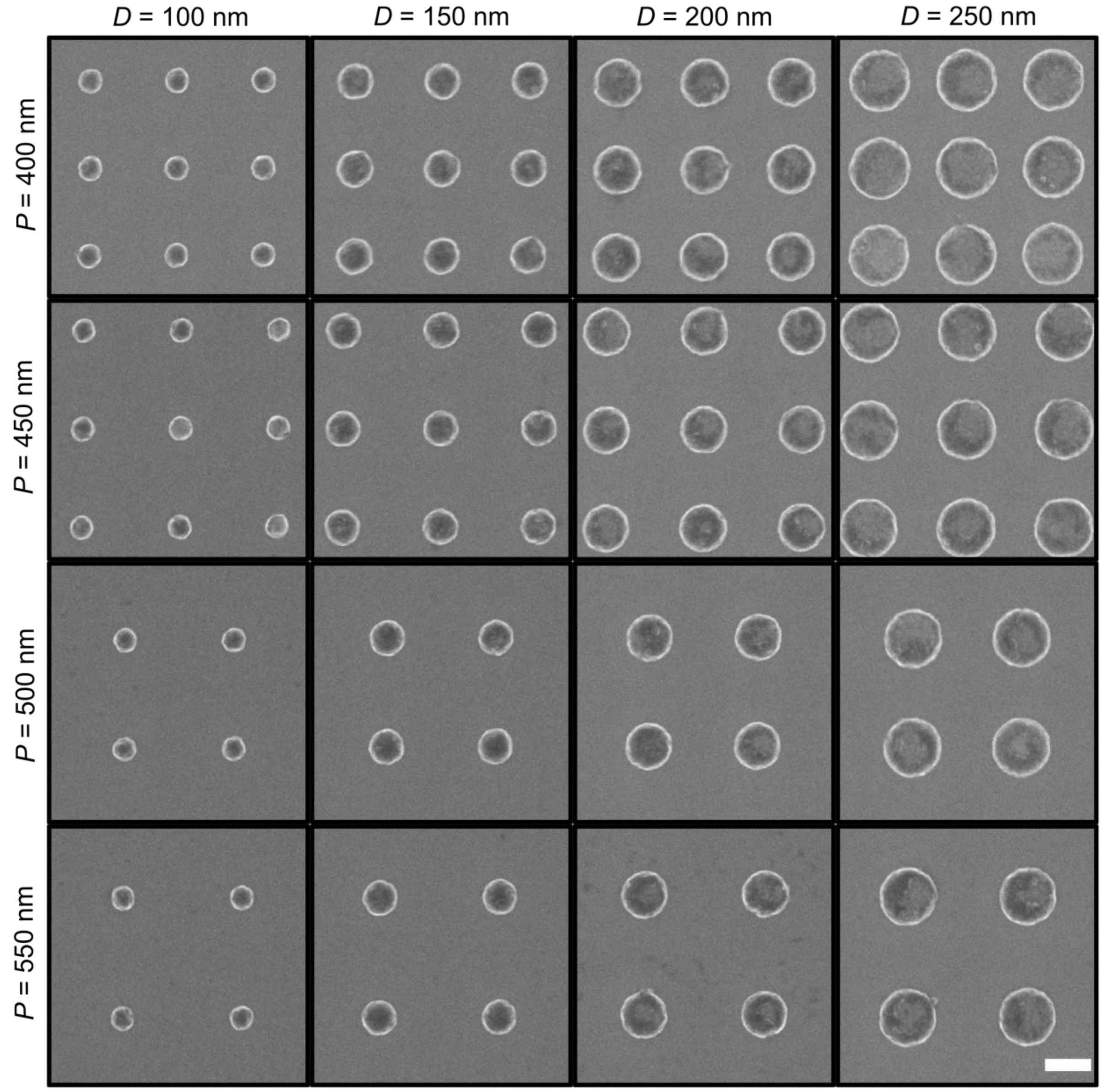

Figure S6. SEM images of $[Co/Gd/Pt]_5$ nanodisk arrays. The scale bar corresponds to 200 nm.

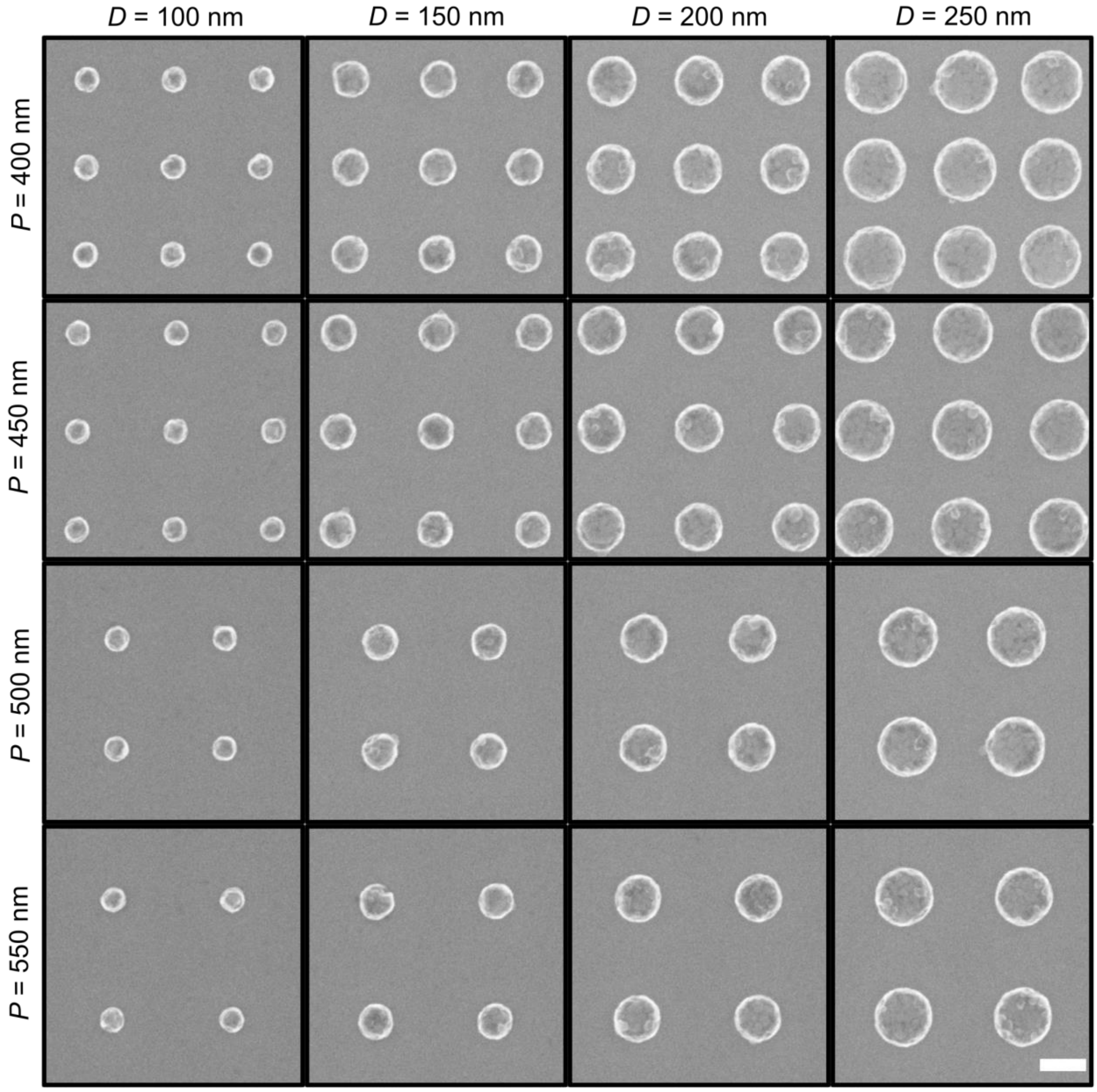

Figure S7. SEM images of $[Co/Gd/Pt]_6$ nanodisk arrays. The scale bar corresponds to 200 nm.

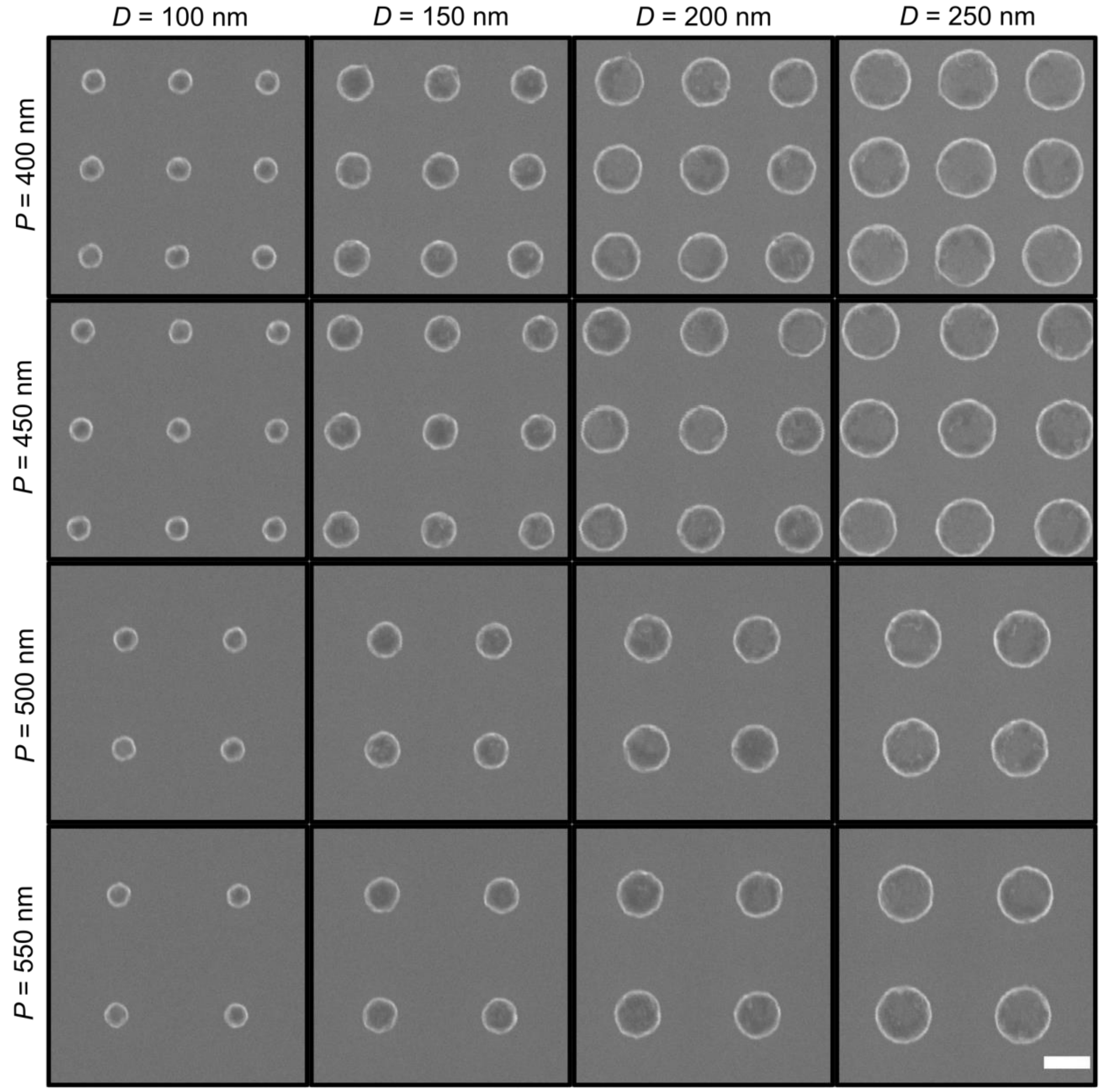

Figure S8. Schematic illustration of the experimental setup for AOS measurements. The microscope contains the following elements: achromatic waveplate, half-wave, quartz-MgF_2 (HWP); a Glan Taylor MGTYA10 polarizer (P_1); two Glan Taylor GT10-A polarizers (P_2 and A_2); silver protected flat mirrors AG20 (M_1); silver protected flat mirrors AG10 (M_2); plano convex lens, N-BK7, f = 1 000 mm (L_1); plano convex lens, N-BK7, f = 50 mm (L_2); EO-5023M MONO USB charge coupled device camera (CCD).

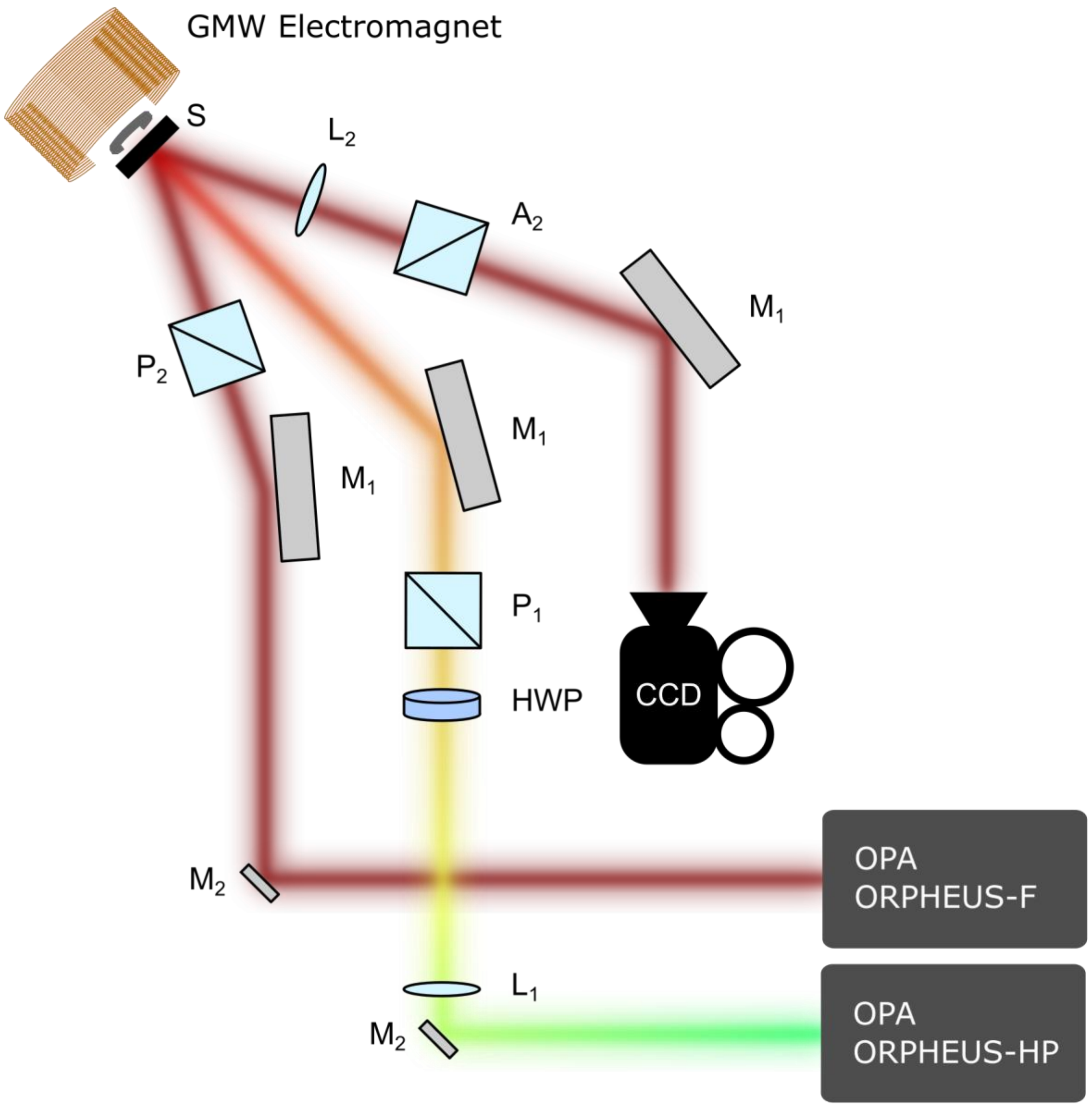

Figure S9. Schematic illustration of the experimental set-up for a) Optical measurement. b) Magneto-optical measurement.

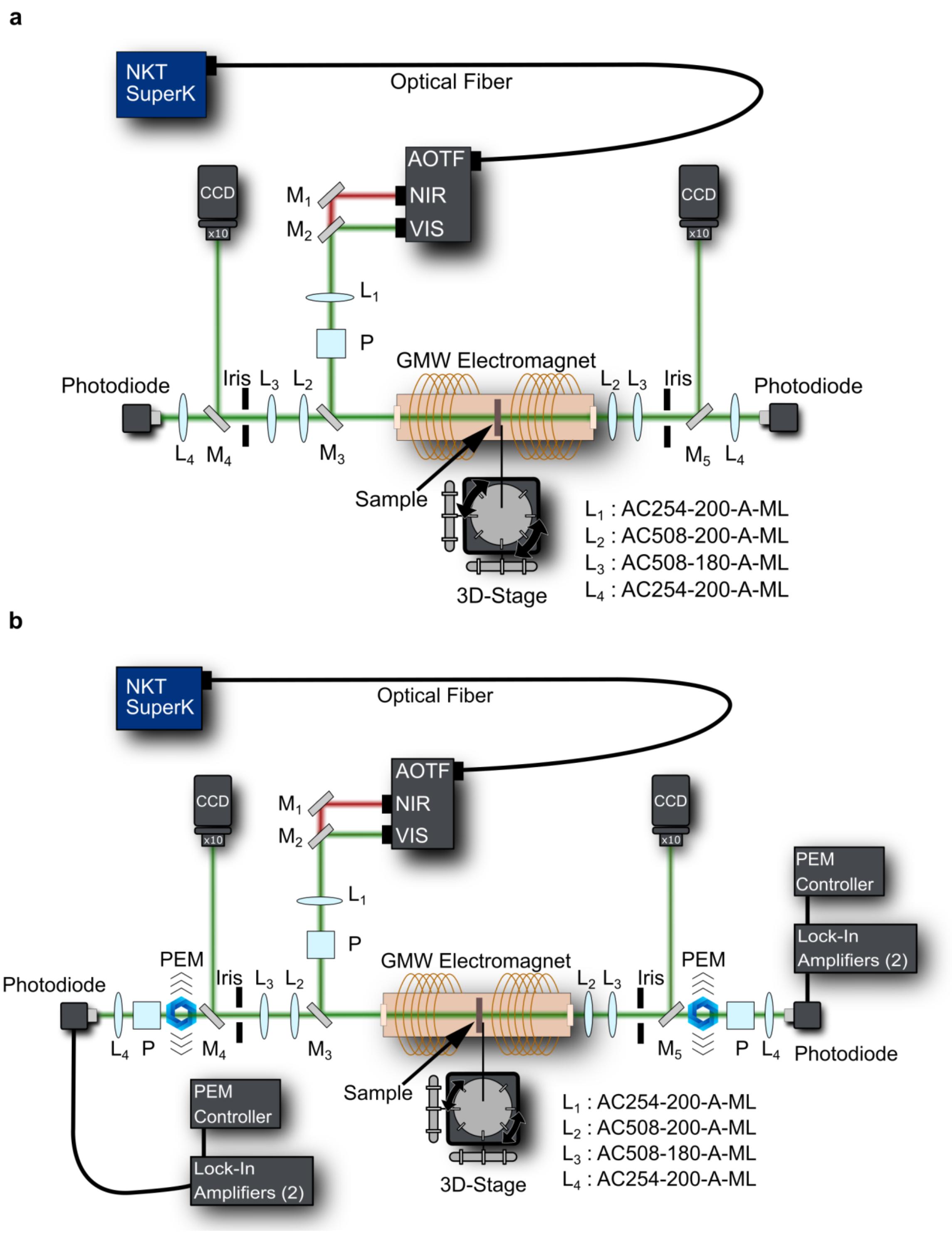

Figure S10. Normalized Kerr rotation as a function of perpendicular magnetic field for $[Co/Gd/Pt]_N$ continuous films with a) N = 1, b) N = 2, c) N = 3, d) N = 4, e) N = 5, f) N = 6.

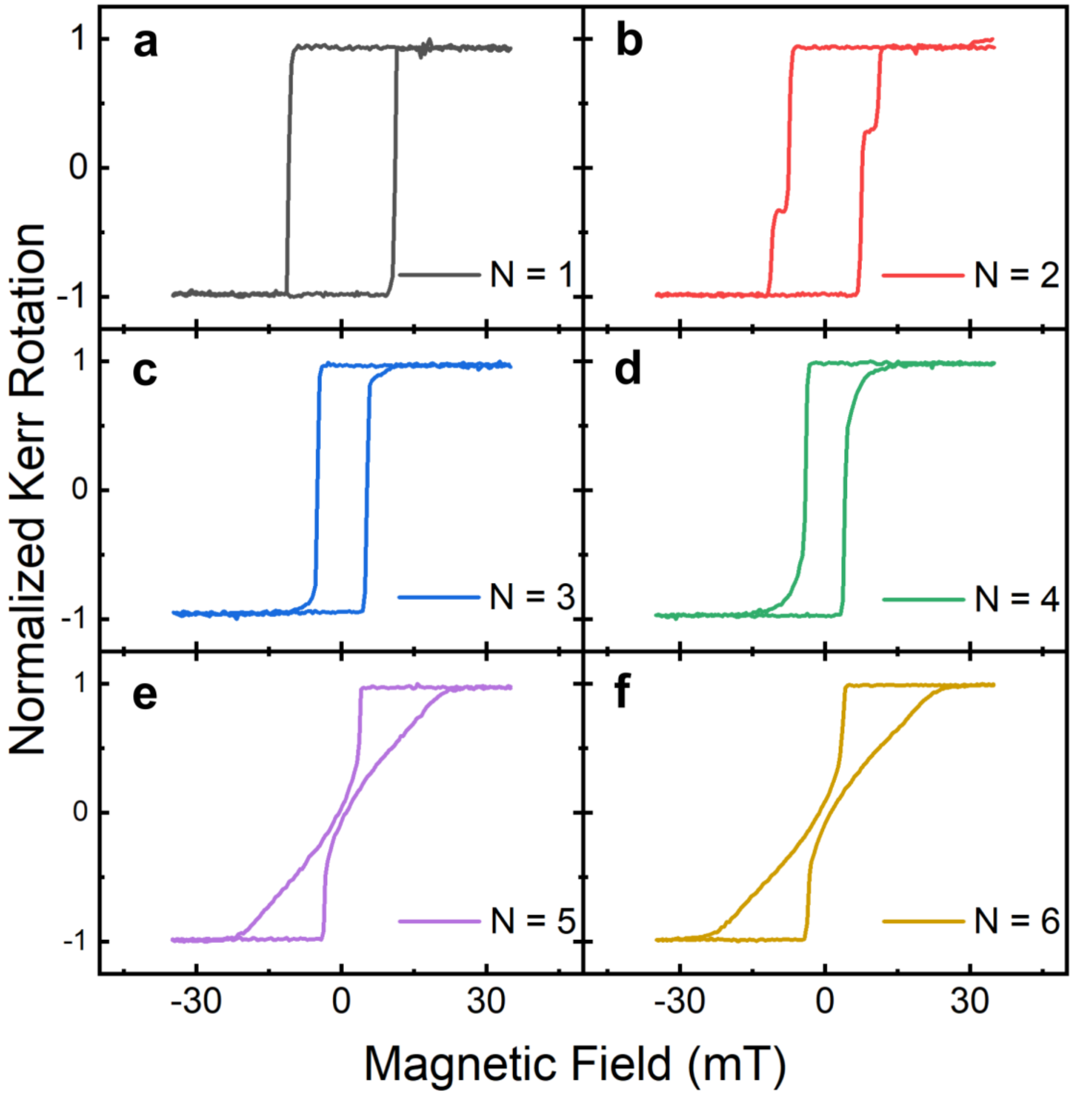

Figure S11. Normalized Faraday rotation as a function of perpendicular magnetic field for $[Co/Gd/Pt]_N$ metasurface with various nanodisk diameters.

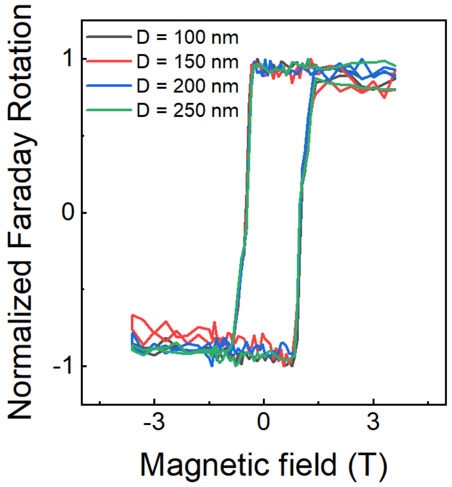

Figure S12. MFM image of $[Co/Gd/Pt]_N$ continuous films for a) N = 5 and b) N = 6. The images are recorded in zero magnetic field.

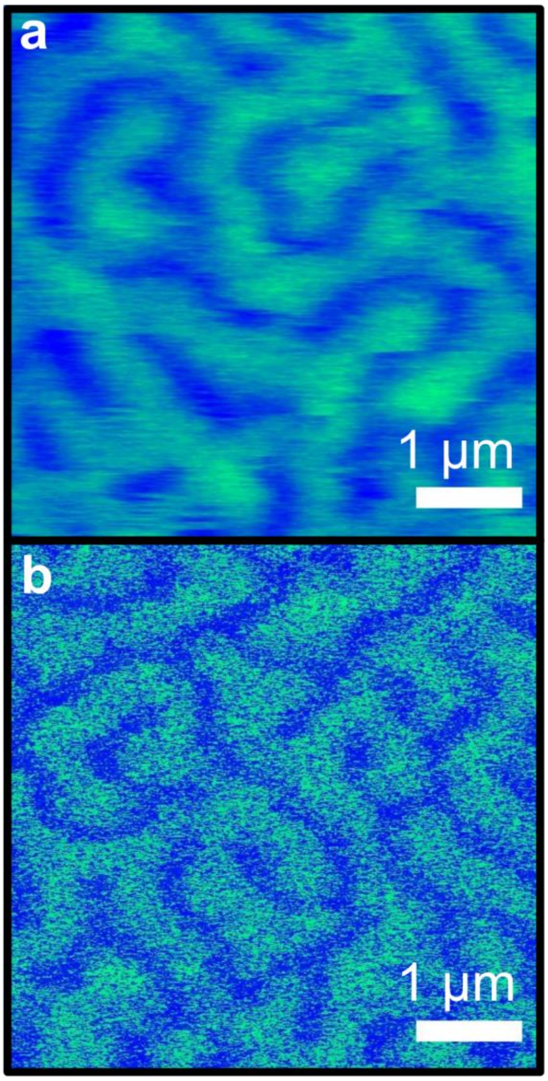

Figure S13. Comparison of single-pulse AO-HIS in a $[Co/Gd/Pt]_2$ film and in patterned $[Co/Gd/Pt]_2$ metasurfaces. a) Ratio $(F_{th-film}/F_{th-array})$ between the AO-HIS threshold fluence measured on a $[Co/Gd/Pt]_2$ film and on corresponding metasurfaces with P = 550 nm and different nanodisk diameters. b) Extinction spectra of the $[Co/Gd/Pt]_2$ metasurfaces normalized to their filling factor and the extinction spectrum of the $[Co/Gd/Pt]_2$ continuous film. c) Simulated normalized extinction spectra for the same metasurfaces.

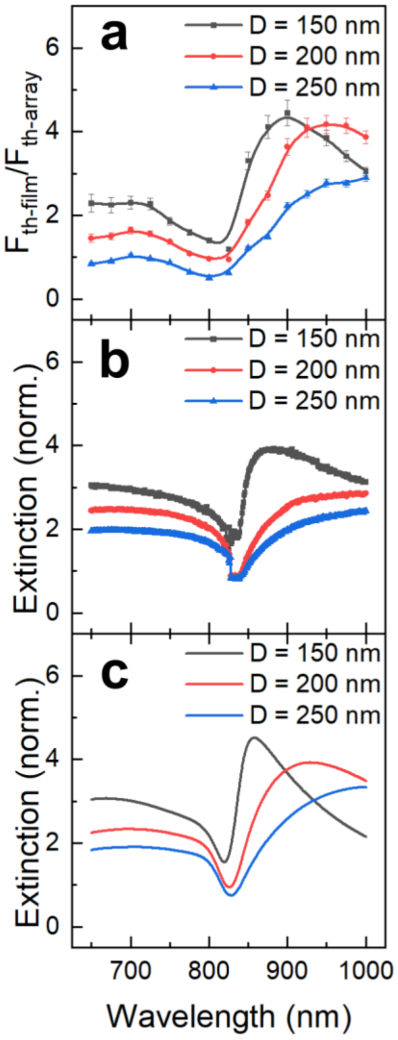

Figure S14. Optical extinction spectra of $[Co/Gd/Pt]_1$ metasurfaces for a) *D* = 100 nm and different array periods, b) *D* = 150 nm and different array periods, c) *D* = 200 nm and different array periods, d) *D* = 250 nm and different array periods.

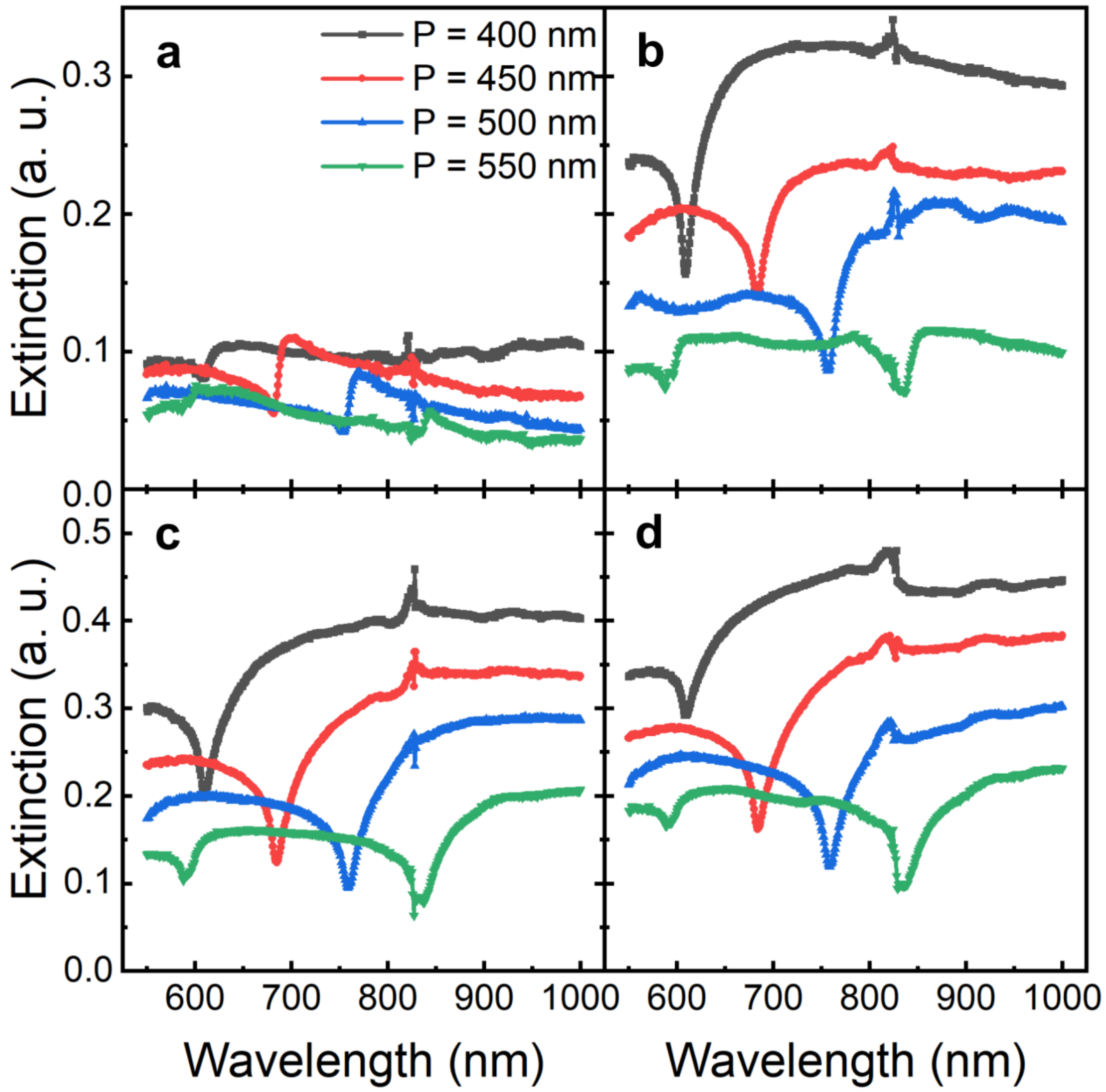

Figure S15. Optical extinction spectra of $[Co/Gd/Pt]_2$ metasurfaces for a) *D* = 100 nm and different array periods, b) *D* = 150 nm and different array periods, c) *D* = 200 nm and different array periods, d) *D* = 250 nm and different array periods.

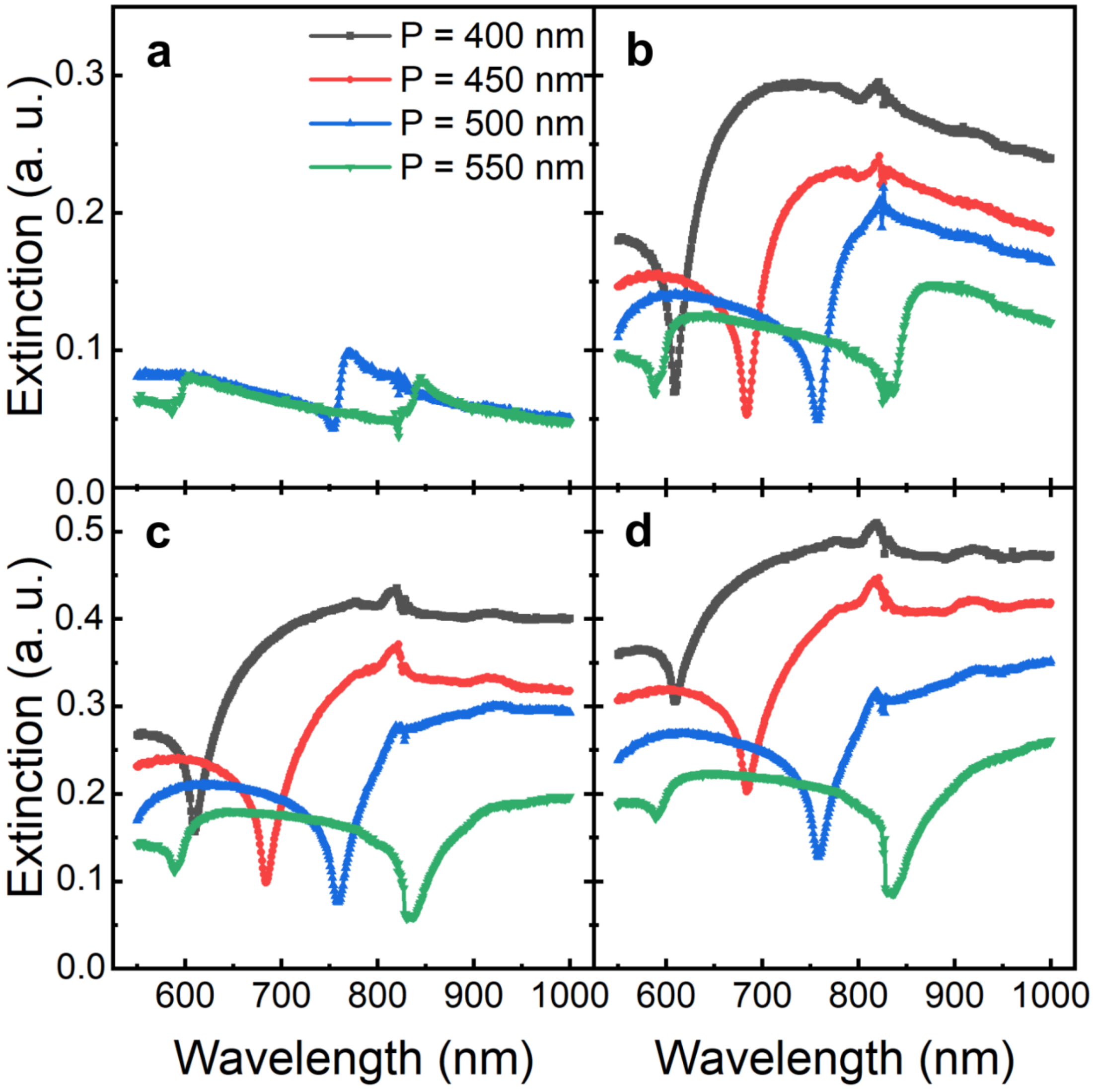

Figure S16. Optical extinction spectra of $[Co/Gd/Pt]_3$ metasurfaces for a) *D* = 100 nm and different array periods, b) *D* = 150 nm and different array periods, c) *D* = 200 nm and different array periods, d) *D* = 250 nm and different array periods.

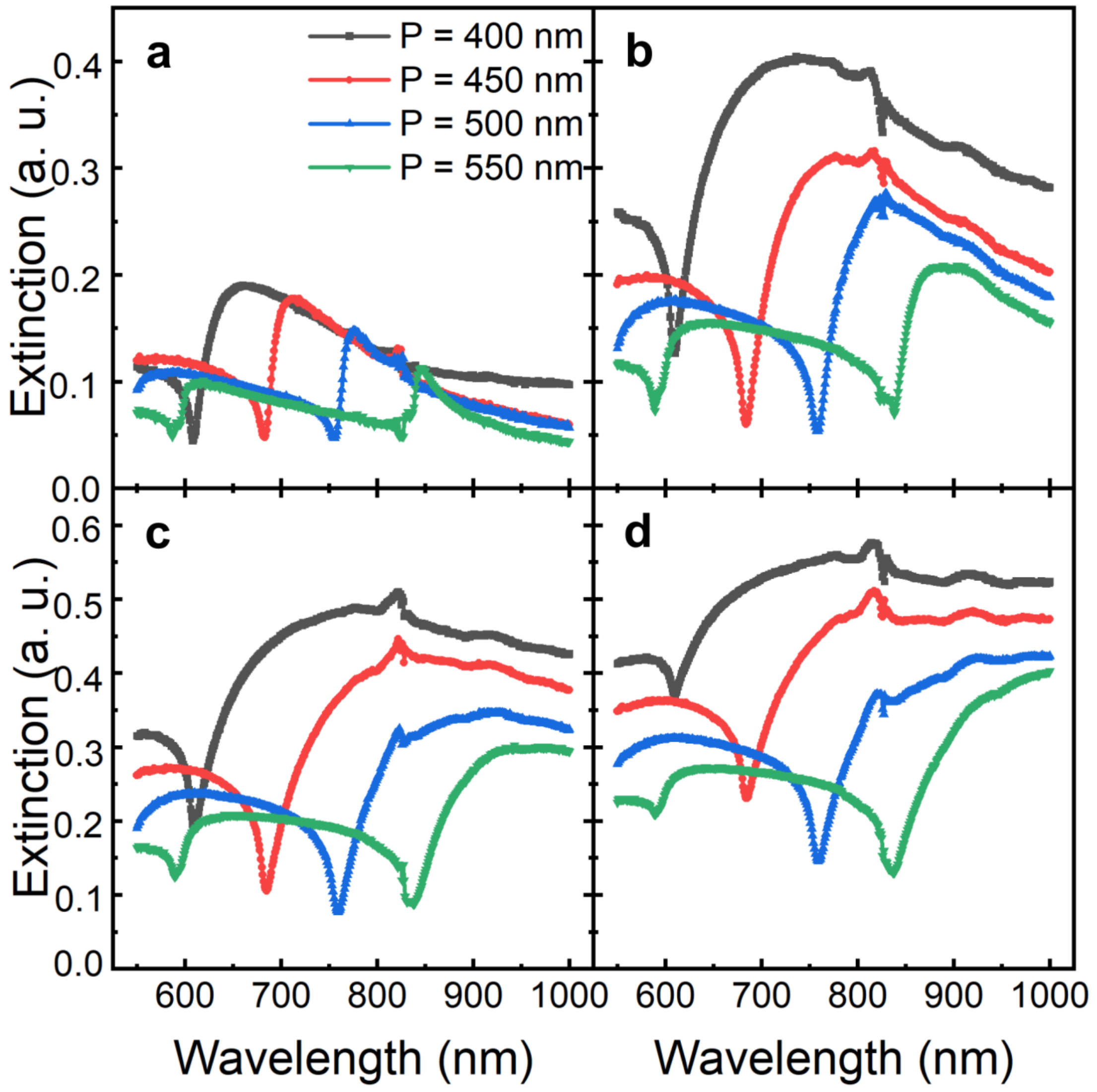

Figure S17. Optical extinction spectra of $[Co/Gd/Pt]_4$ metasurfaces for a) *D* = 100 nm and different array periods, b) *D* = 150 nm and different array periods, c) *D* = 200 nm and different array periods, d) *D* = 250 nm and different array periods.

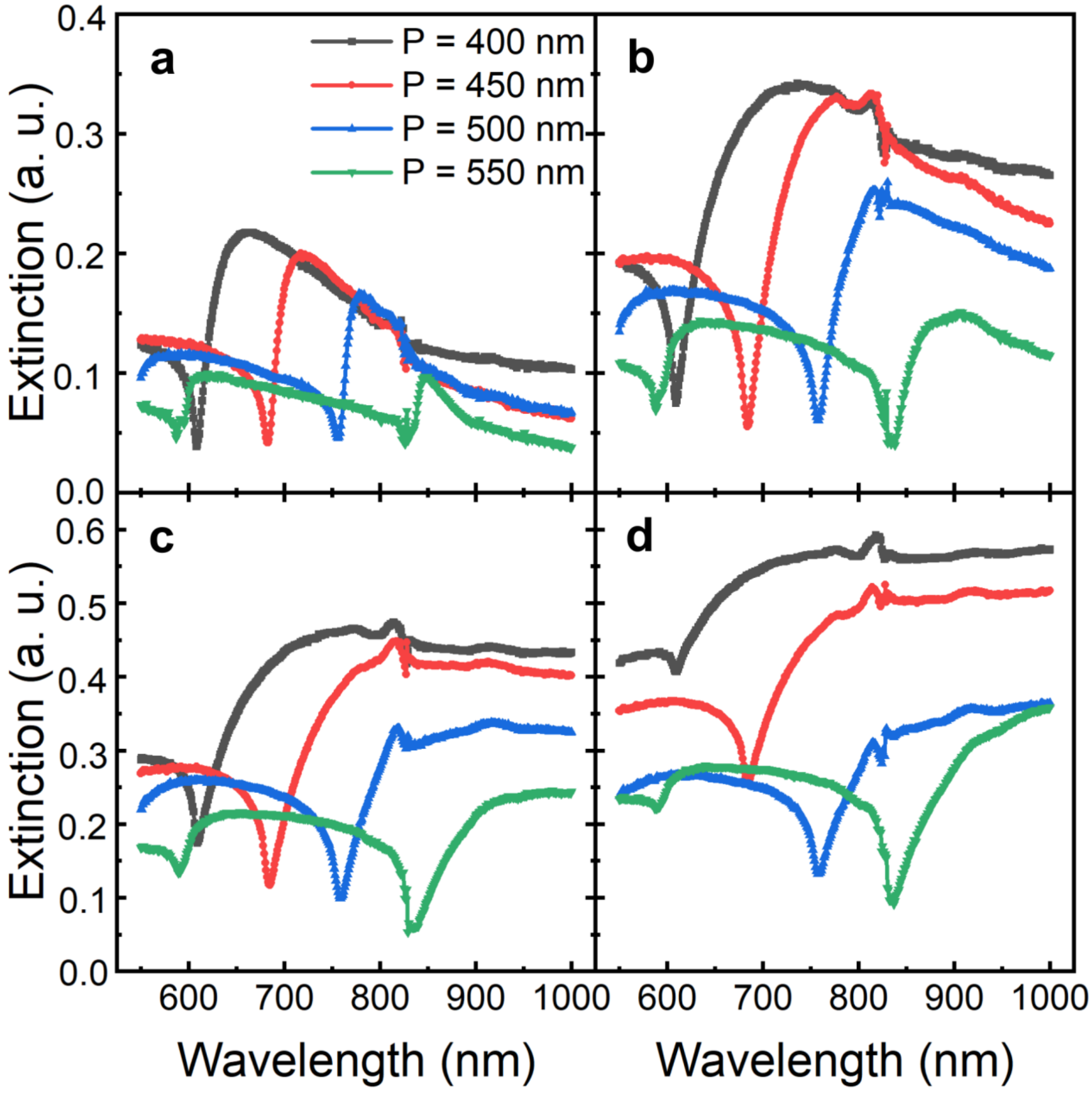

Figure S18. Optical extinction spectra of $[Co/Gd/Pt]_5$ metasurfaces for a) D = 100 nm and different array periods, b) D = 150 nm and different array periods, c) D = 200 nm and different array periods, d) D = 250 nm and different array periods.

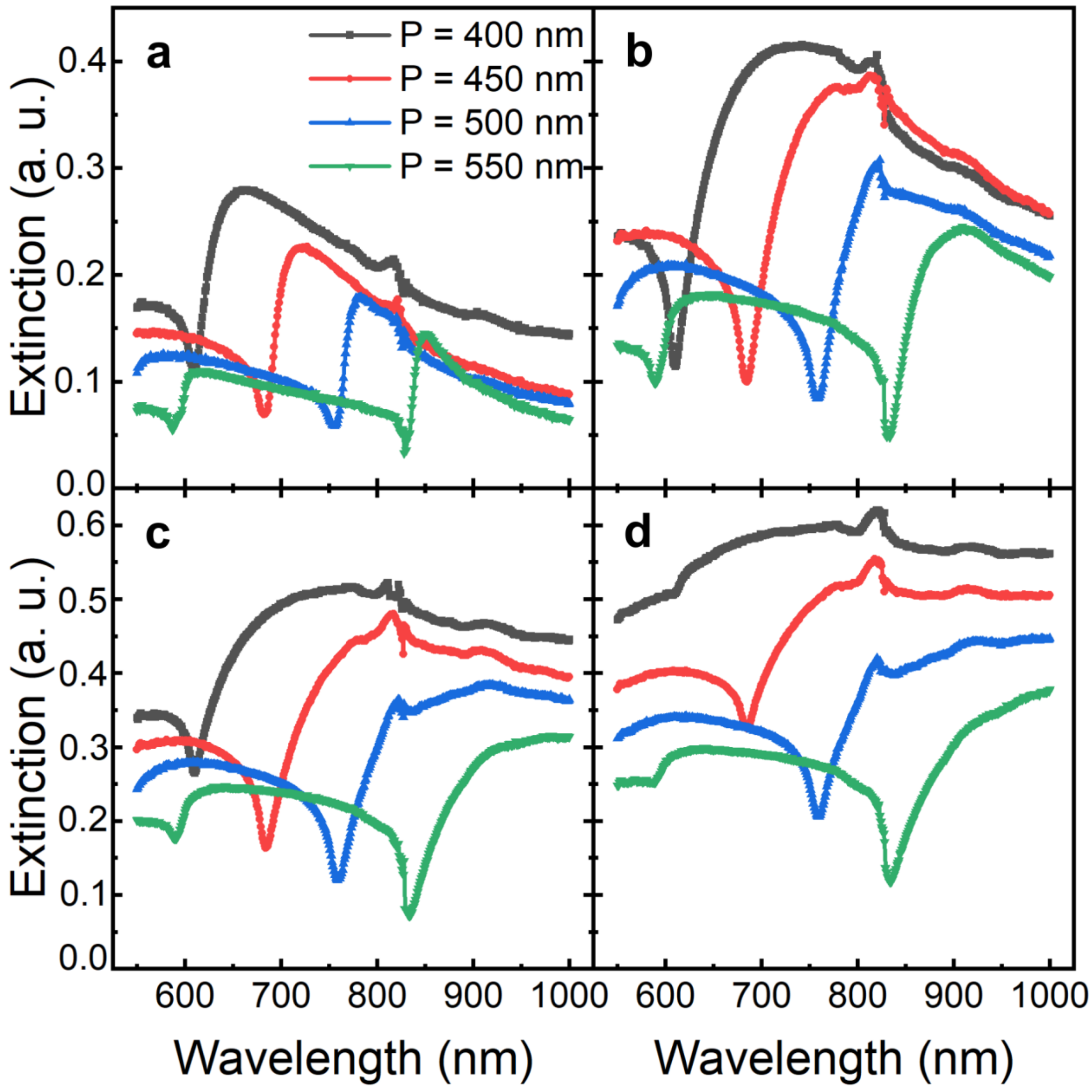

Figure S19. Optical extinction spectra of $[Co/Gd/Pt]_6$ metasurfaces for a) *D* = 100 nm and different array periods, b) *D* = 150 nm and different array periods, c) *D* = 200 nm and different array periods, d) *D* = 250 nm and different array periods.

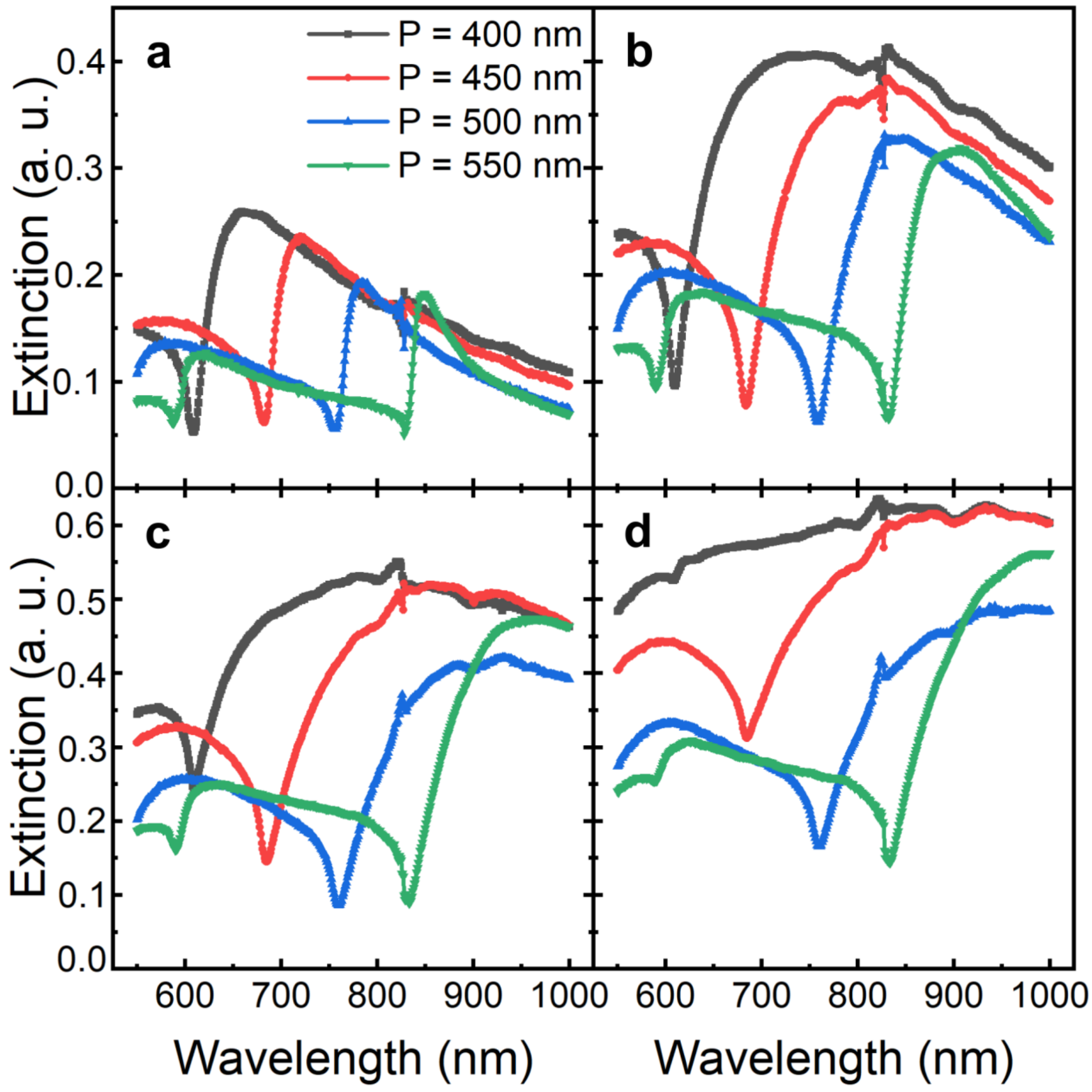

Figure S20. Optical constants measured at room temperature of [Pt(1)/[Pt(3)/Gd(2)/Co(1)]$_N$/Pt(5)/Ta(5) film for a) N = 1, b) N = 2, c) N = 3, d) N = 4, e) N = 5, f) N = 6

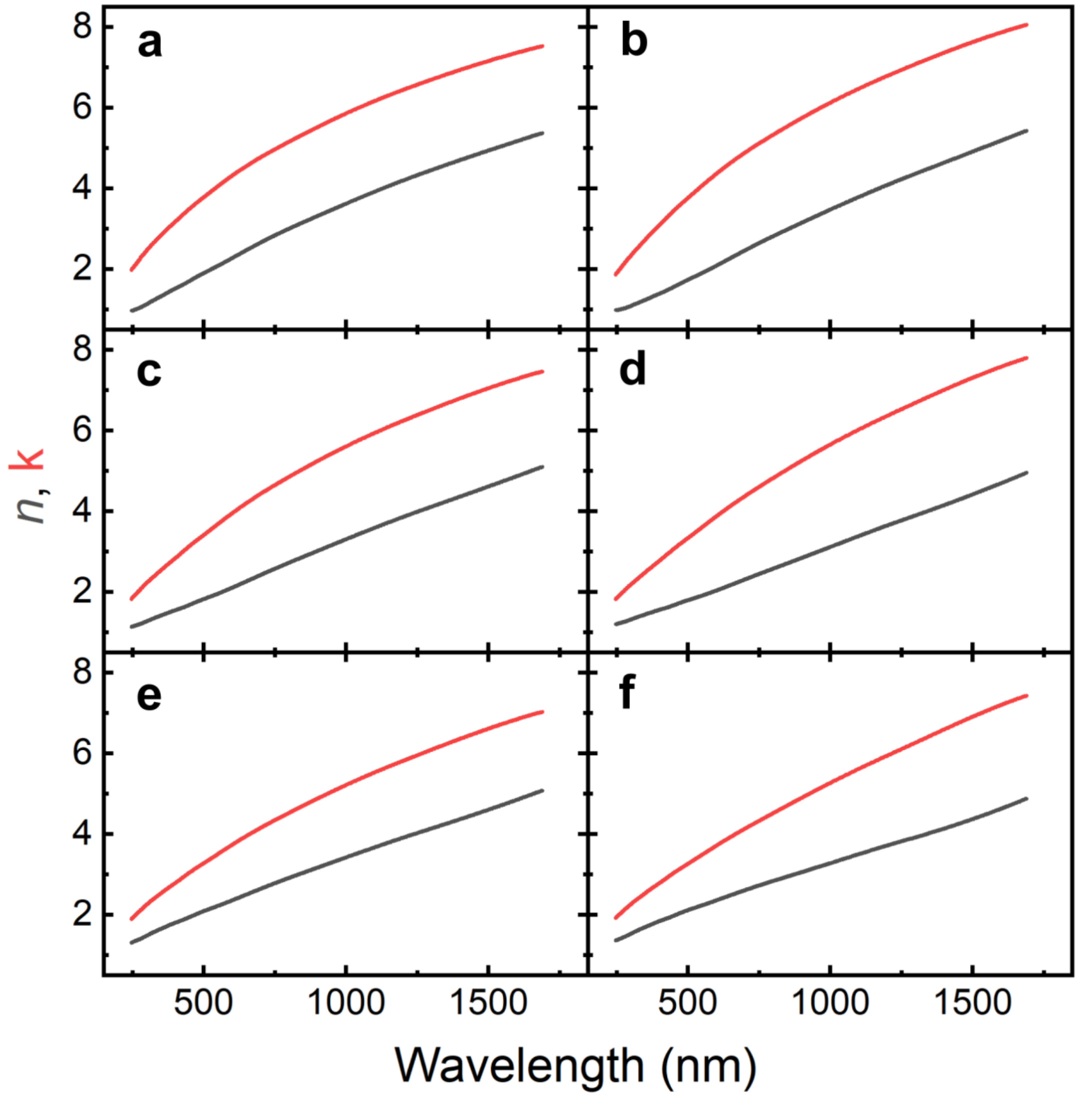

Figure S21. Toggle switching on $[Co/Gd/Pt]_2$ continuous film with a fluence F = 3.02 mJ/cm² at 650 nm. Red and blue colors indicate magnetization pointing up and down, respectively. The scale bar corresponds to 50 μm.

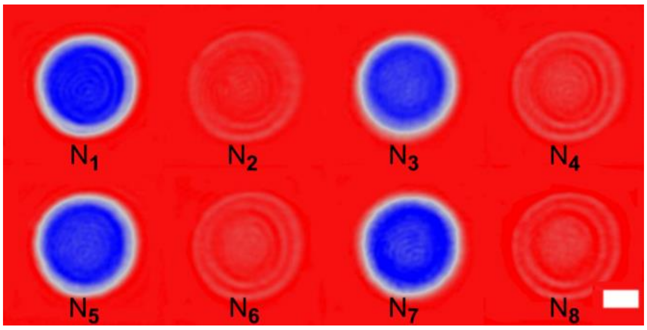

Figure S22. Repetitive switching of single-pulse AO-HIS in a $[Co/Gd/Pt]_2$ metasurface with D = 200 nm and P = 500 nm for a fluence of 1.89 mJ/cm² at 650 nm. Red and blue colors indicate magnetization pointing up and down, respectively. The scale bar corresponds to 50 μm.

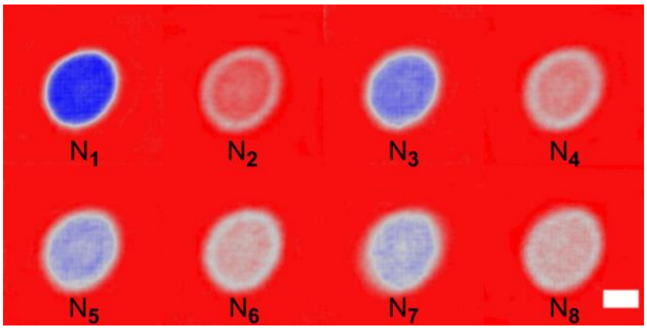

Figure S23. Repetitive switching of single-pulse AO-HIS in a $[Co/Gd/Pt]_2$ metasurface with D = 250 nm and P = 500 nm for a fluence of 2.77 mJ/cm² at 650 nm. Red and blue colors indicate magnetization pointing up and down, respectively. The scale bar corresponds to 50 µm.

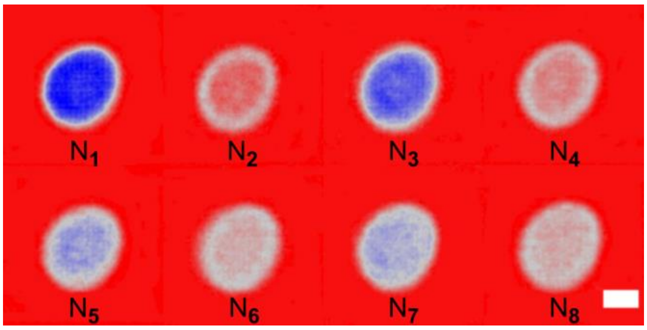

Figure S24. Faraday angle spectra of $[Co/Gd/Pt]_1$ metasurfaces for a) D = 100 nm and different array periods, b) D = 150 nm and different array periods, c) D = 200 nm and different array periods, d) D = 250 nm and different array periods.

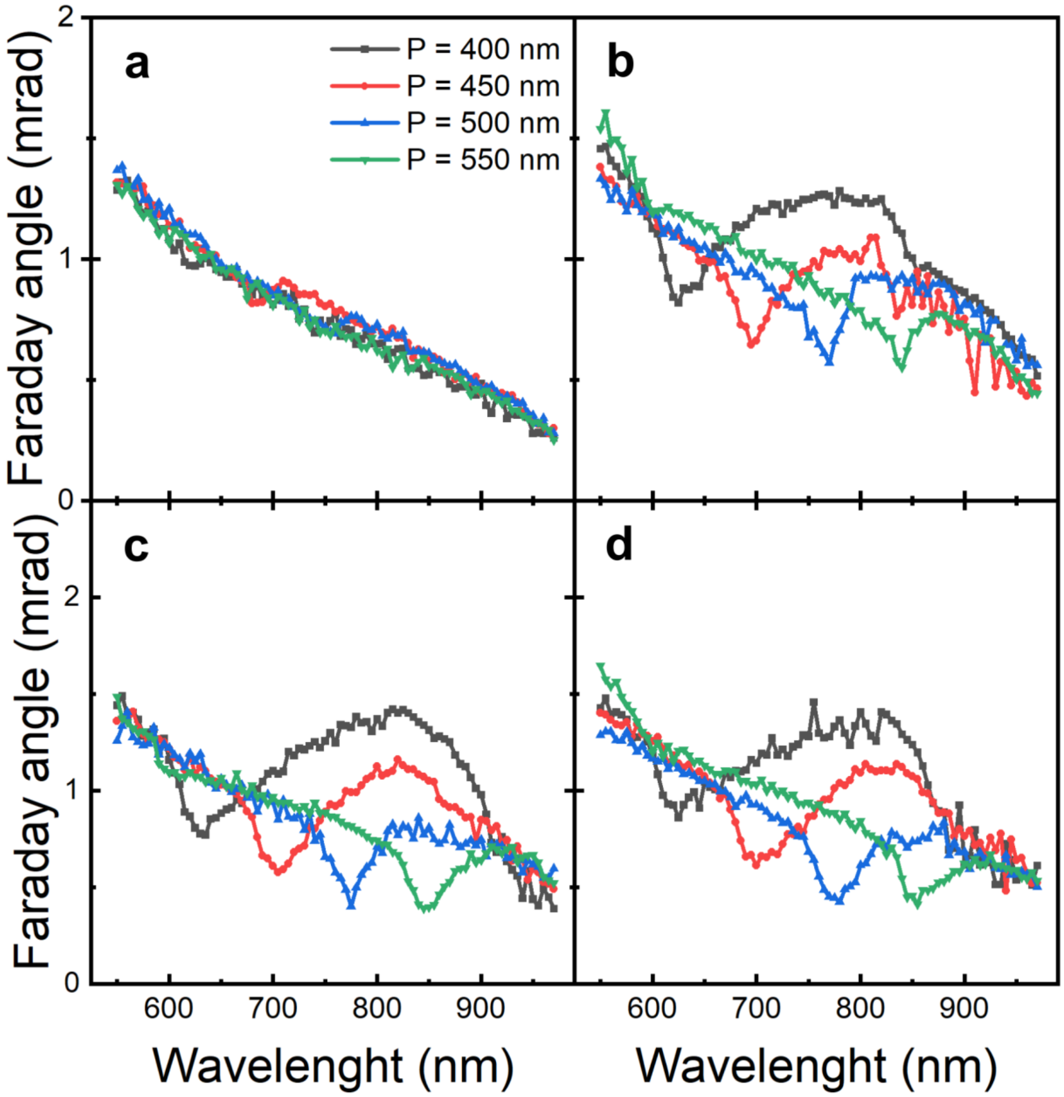

Figure S25. Faraday angle spectra of $[Co/Gd/Pt]_2$ metasurfaces for a) *D* = 100 nm and different array periods, b) *D* = 150 nm and different array periods, c) *D* = 200 nm and different array periods, d) *D* = 250 nm and different array periods.

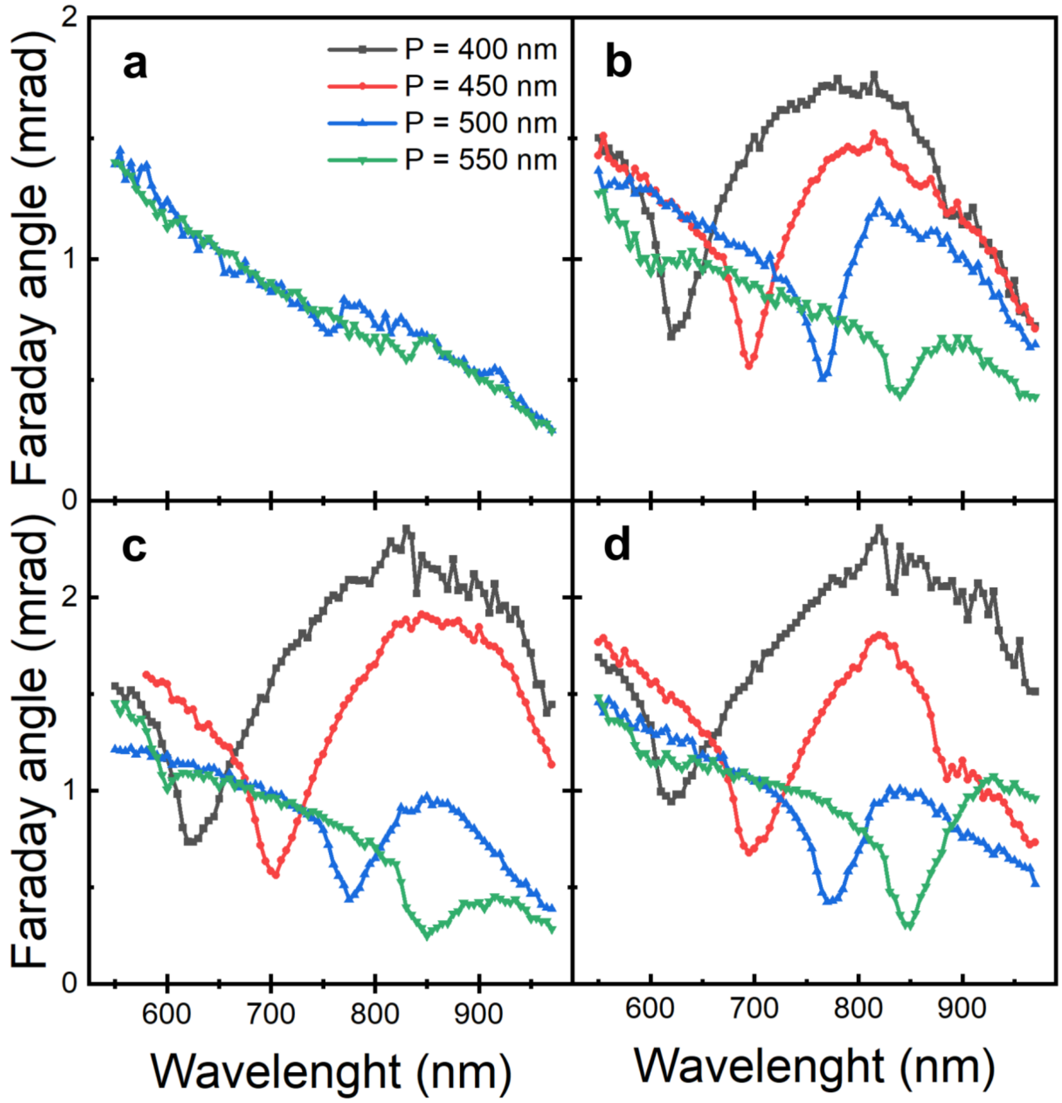

Figure S26. Faraday angle spectra of $[Co/Gd/Pt]_3$ metasurfaces for a) *D* = 100 nm and different array periods, b) *D* = 150 nm and different array periods, c) *D* = 200 nm and different array periods, d) *D* = 250 nm and different array periods.

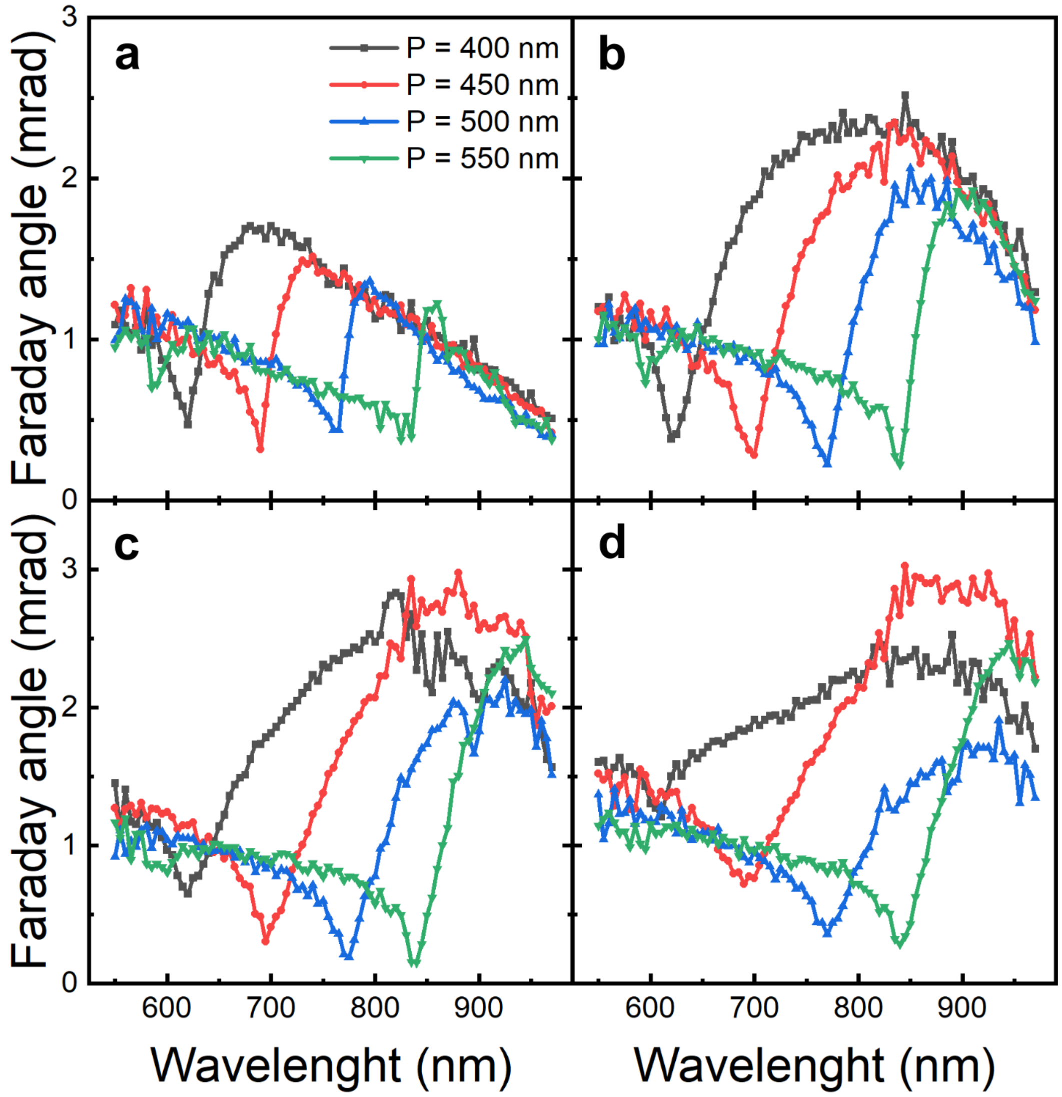

Figure S27. Faraday angle spectra of [Co/Gd/Pt]$_4$ metasurfaces for a) *D* = 100 nm and different array periods, b) *D* = 150 nm and different array periods, c) *D* = 200 nm and different array periods, d) *D* = 250 nm and different array periods.

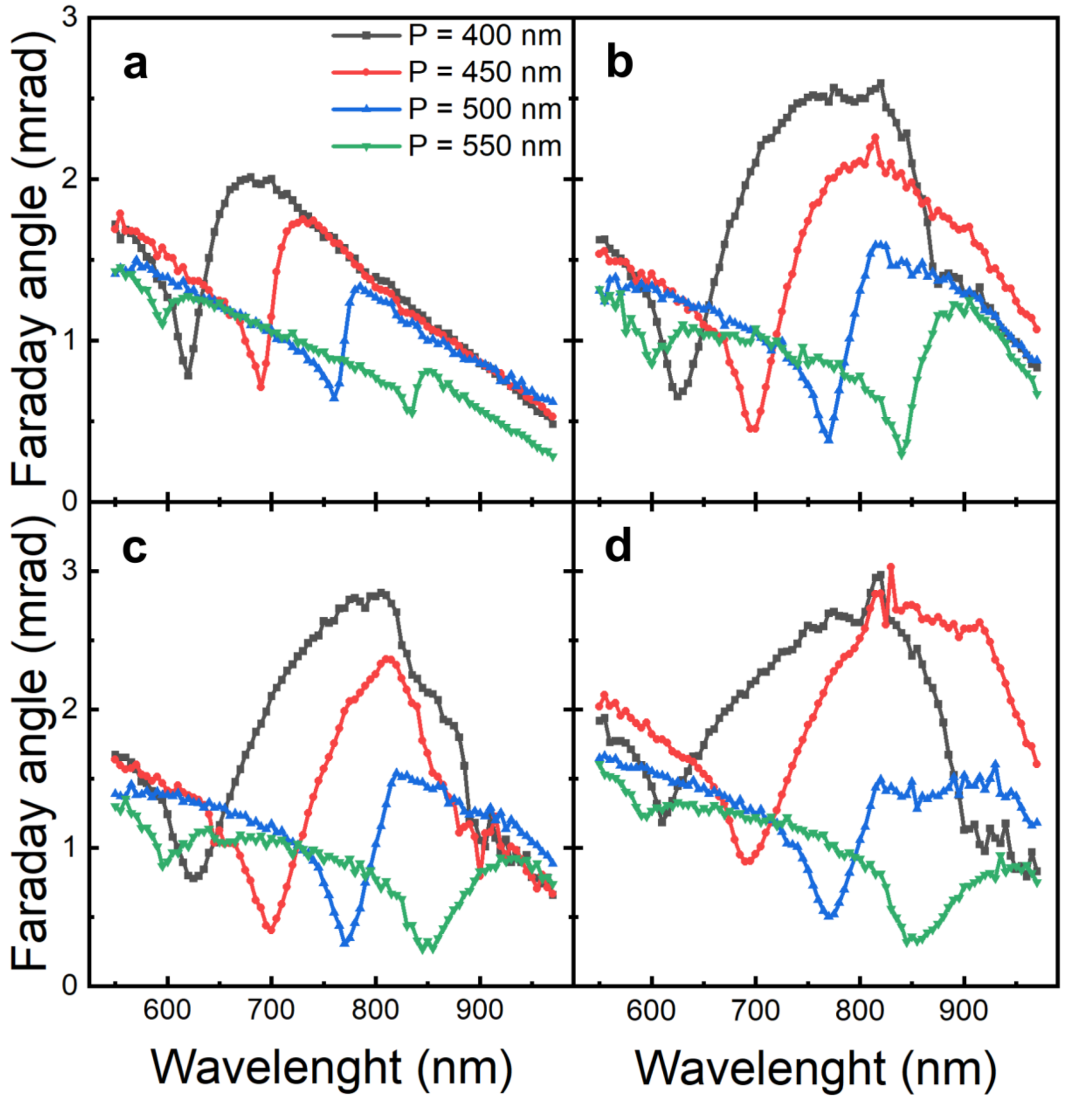

Figure S28. Faraday angle spectra of $[Co/Gd/Pt]_5$ metasurfaces for a) D = 100 nm and different array periods, b) D = 150 nm and different array periods, c) D = 200 nm and different array periods, d) D = 250 nm and different array periods.

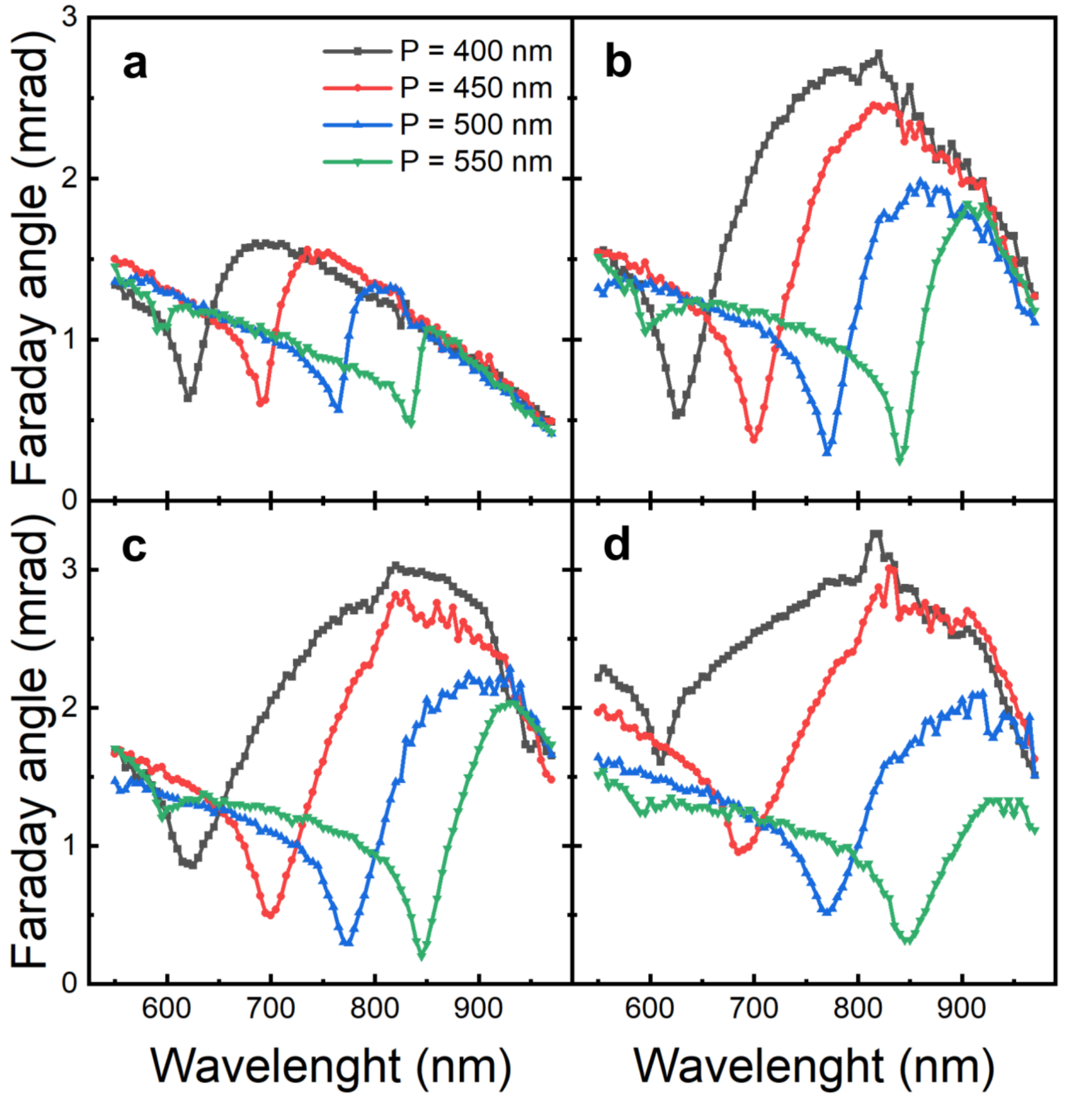

Figure S29. Faraday angle spectra of [Co/Gd/Pt]$_6$ metasurfaces for a) *D* = 100 nm and different array periods, b) *D* = 150 nm and different array periods, c) *D* = 200 nm and different array periods, d) *D* = 250 nm and different array periods.

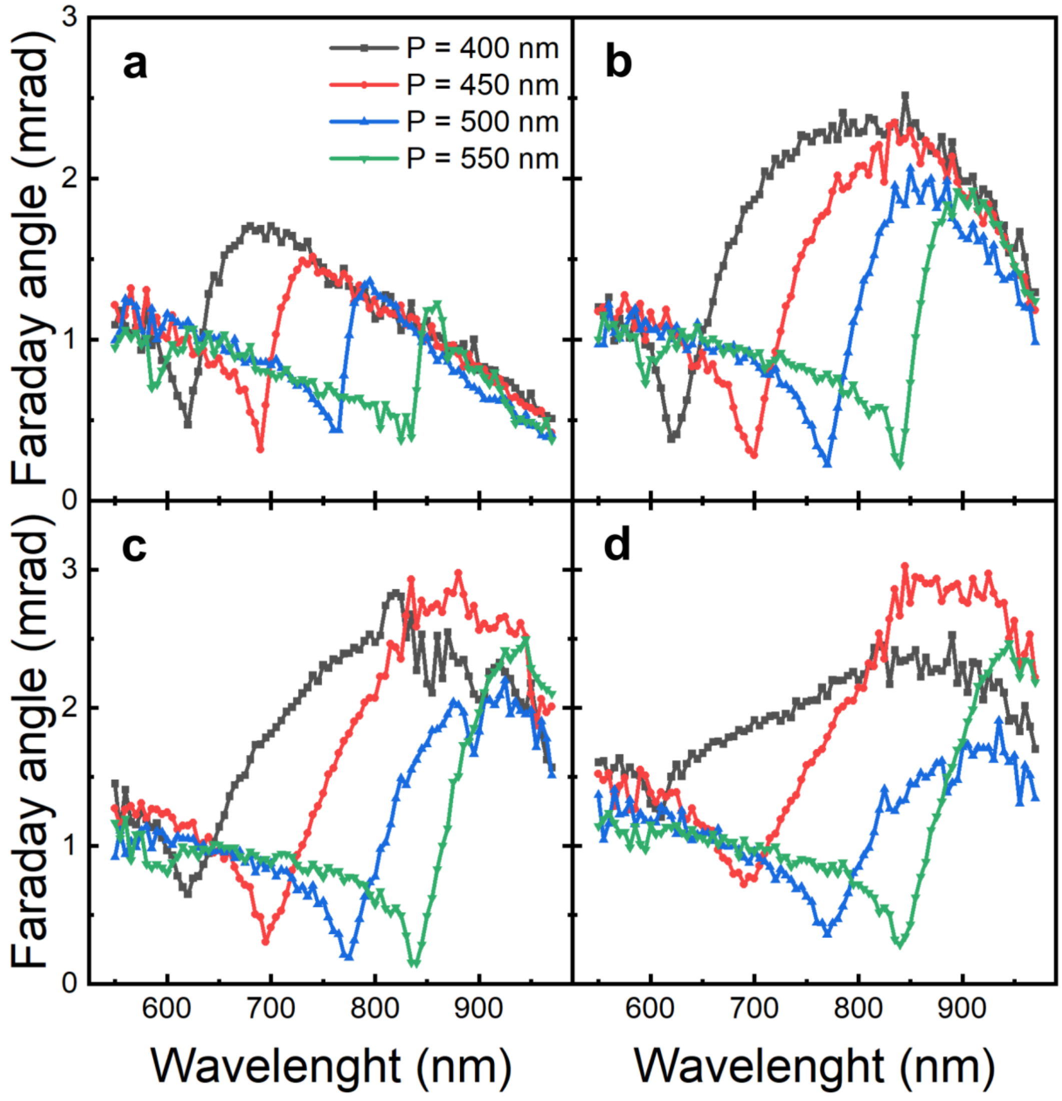

Figure S30. a) Optical extinction and b) Faraday angle spectra of $[Co/Gd/Pt]_N$ films.

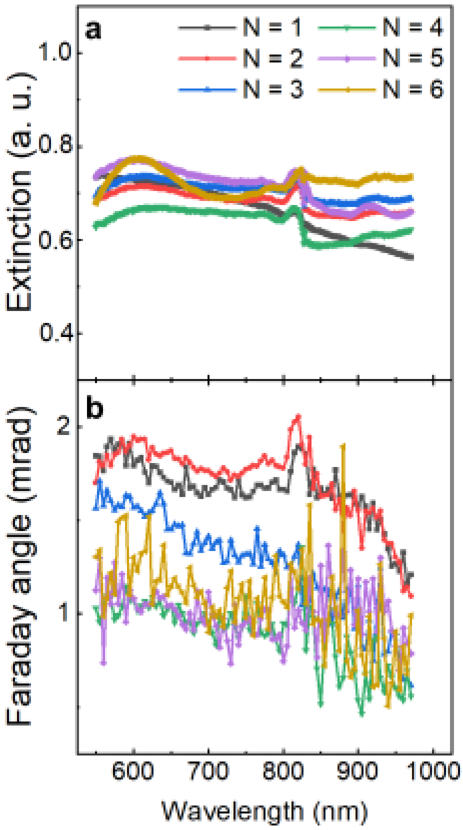

Figure S31. SLR enhanced Faraday angle for $[Co/Gd/Pt]_N$ for a) $N = 1$, b) $N = 2$, c) $N = 3$, d) $N = 4$, e) $N = 5$, f) $N = 6$.

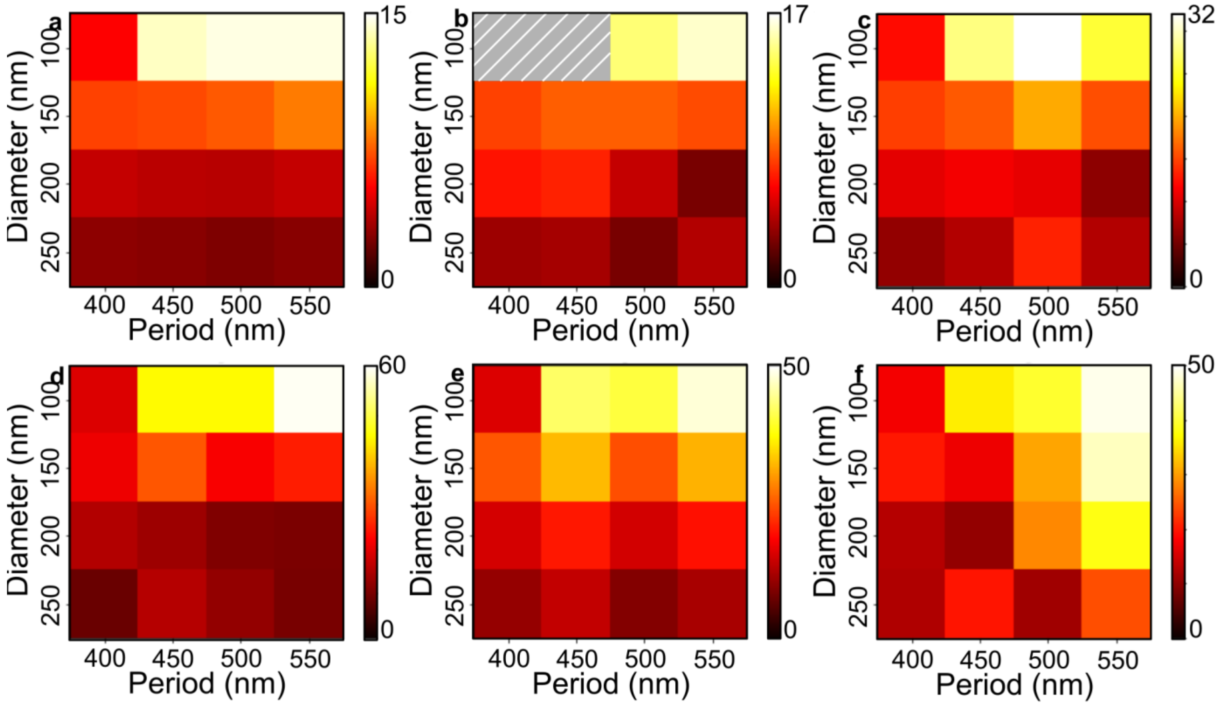

Figure S32. Magneto-optical contrast of $[Co/Gd/Pt]_3$ metasurfaces for a) D = 200 nm. b) D = 250 nm.

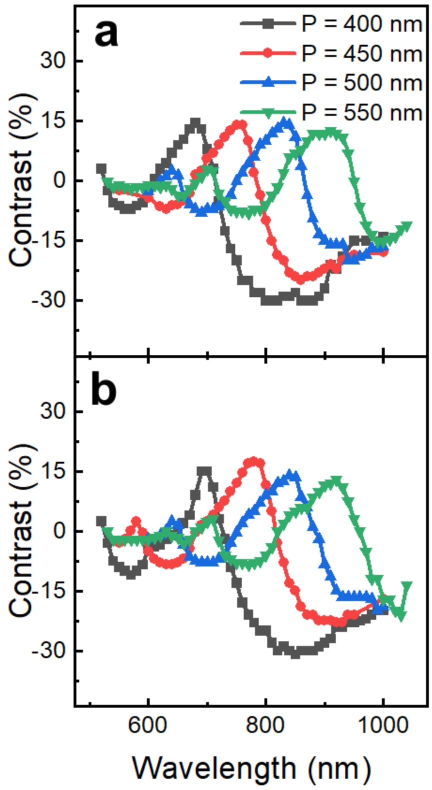

Supplement: Supplementary file 1 — Supporting Information [file ADVS-10-2204683-s001.pdf]
